# Supplementary figures and images for: Overcoming chemotherapy resistance in low-grade gliomas: A computational approach
Source: PLoS Comput Biol. 2023 Nov 20;19(11):e1011208. doi: 10.1371/journal.pcbi.1011208 (PMC10695391; doi:10.1371/journal.pcbi.1011208)

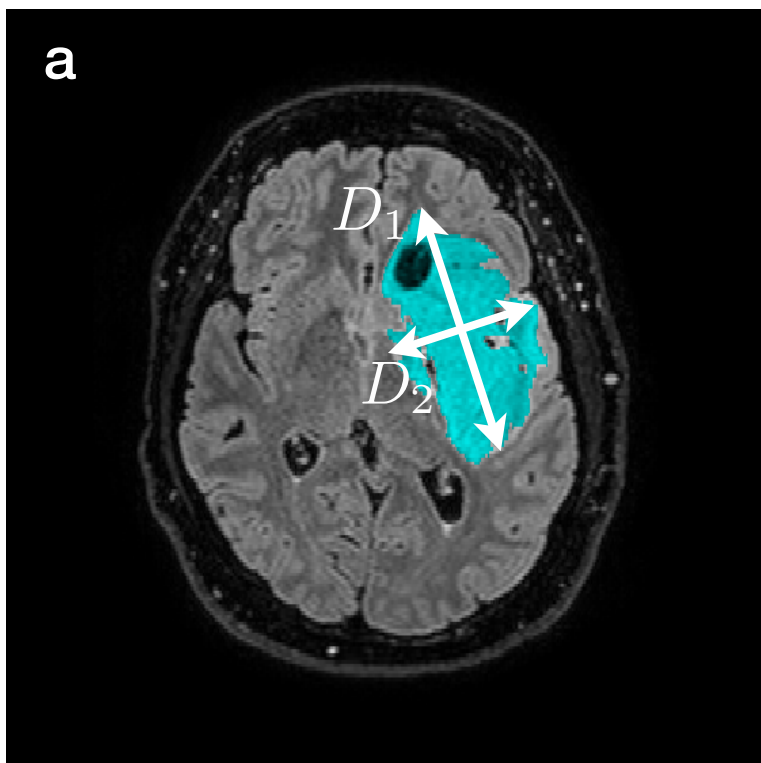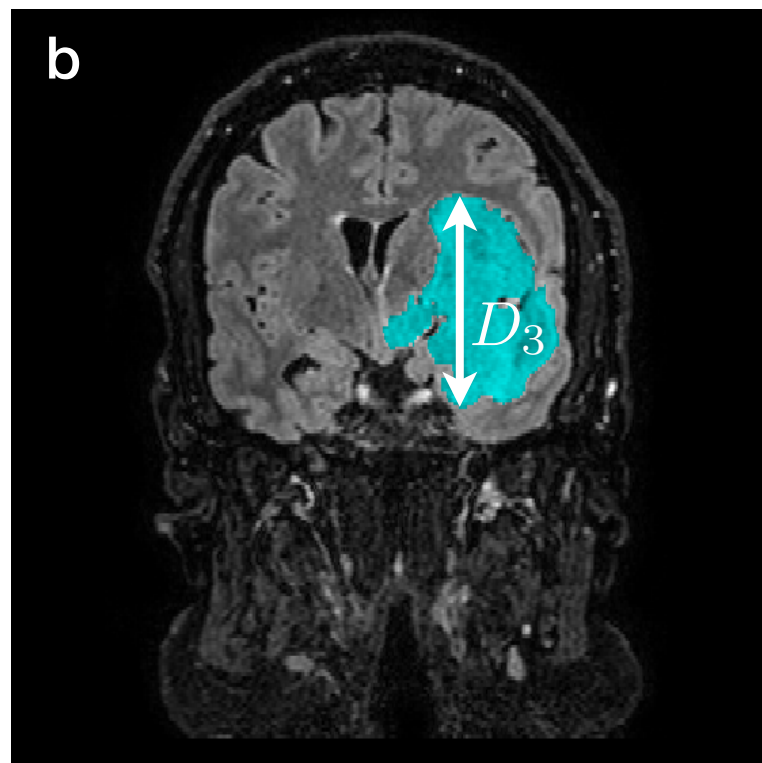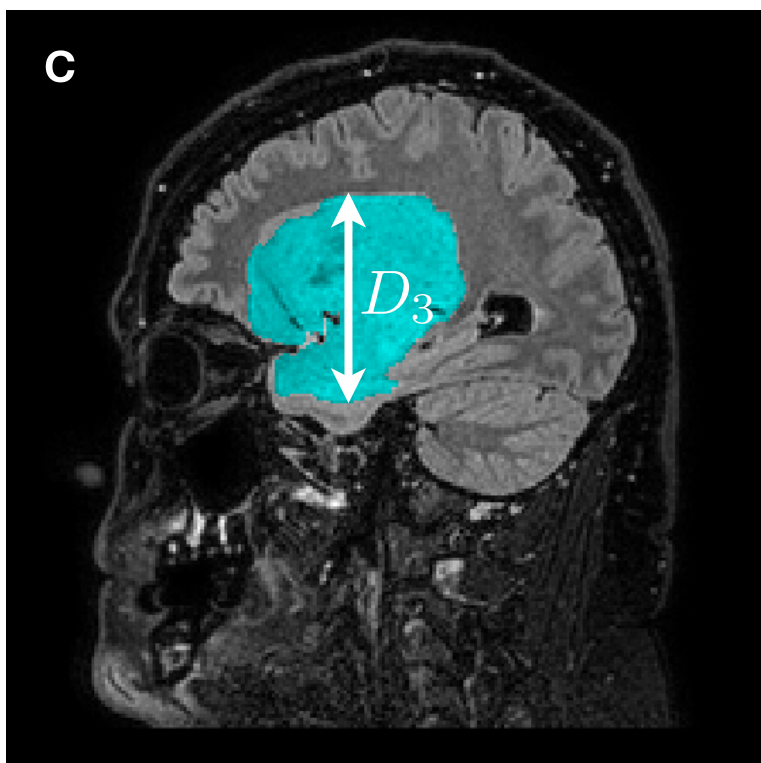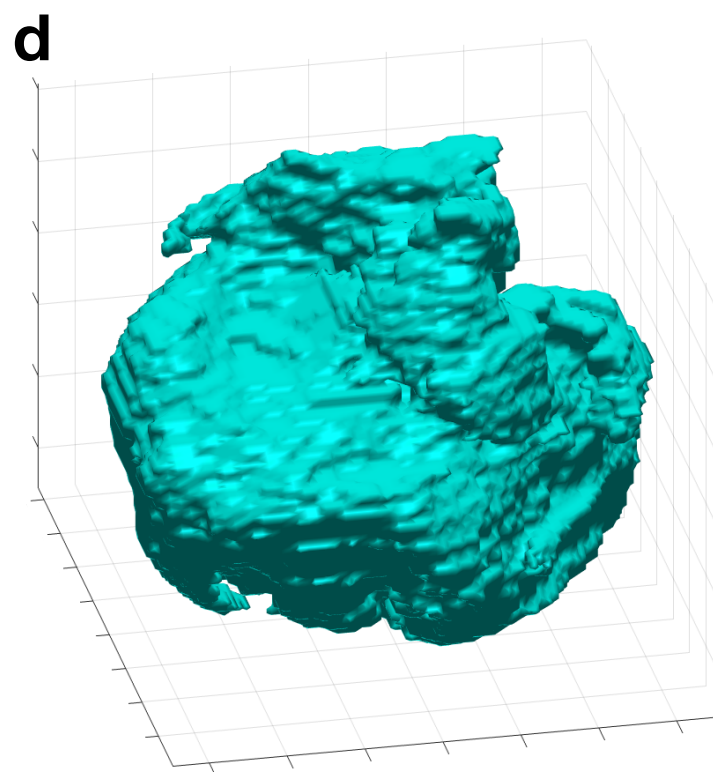

Supplement: S1 Fig — Axial (A), coronal (B) and sagittal (C) slices of FLAIR 3D MRI study of patient 7 at the time of diagnosis. The blue mask indicates the area delineated semi-automatically using a gray-level threshold. Above it, the white arrows indicate the measure of the diameters used for the ellipsoidal approximation of tumor volume. (D) 3D reconstruction of the segmented tumor. (PDF) [file pcbi.1011208.s001.pdf]

**A.**

Initial volume distribution

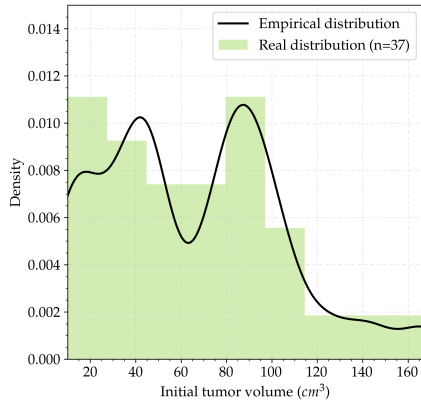**B.**

TMZ cycles distribution

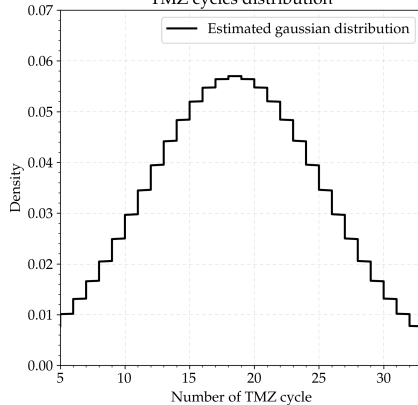**C.**

Fatal volume distribution

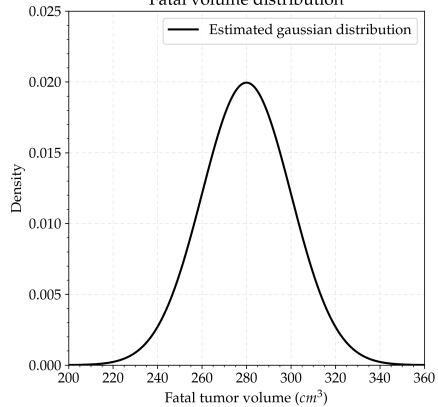

Supplement: S2 Fig — Statistical distribution of initial tumor volume, number of TMZ doses and fatal volume used to construct the virtual patients. (PDF) [file pcbi.1011208.s002.pdf]

A.

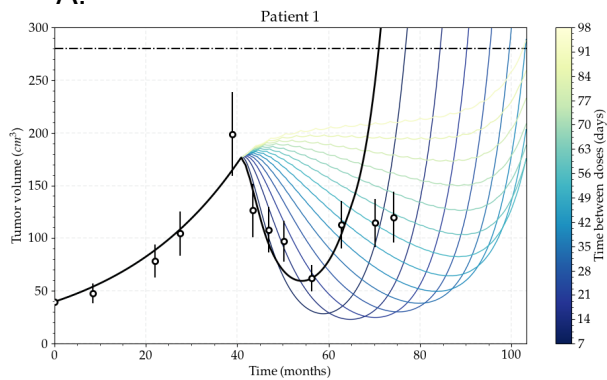

B.

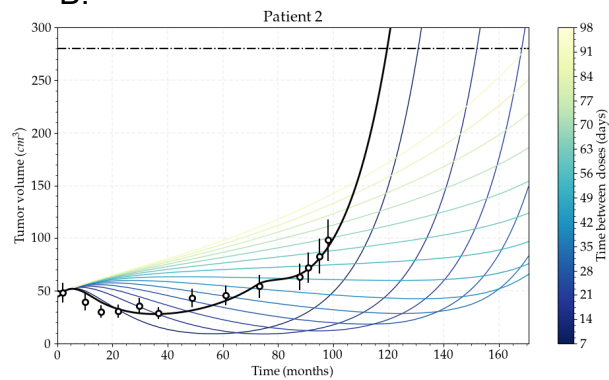

C.

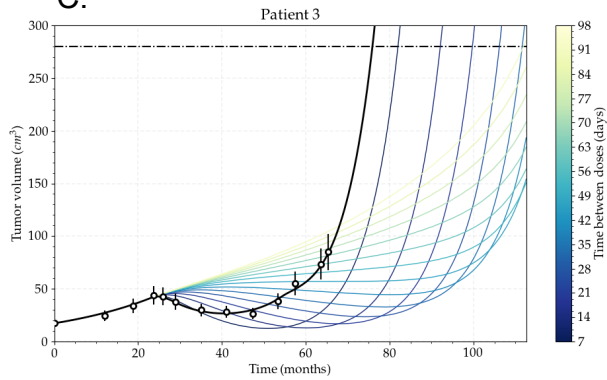

D.

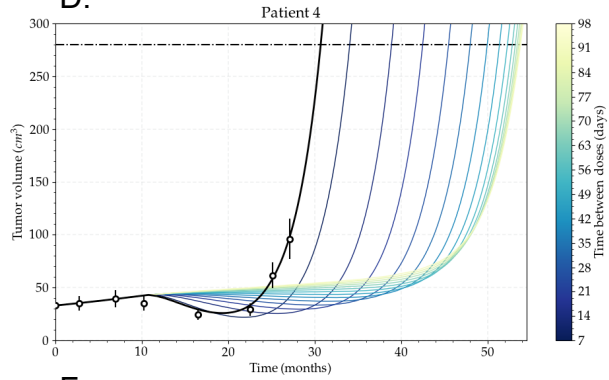

E.

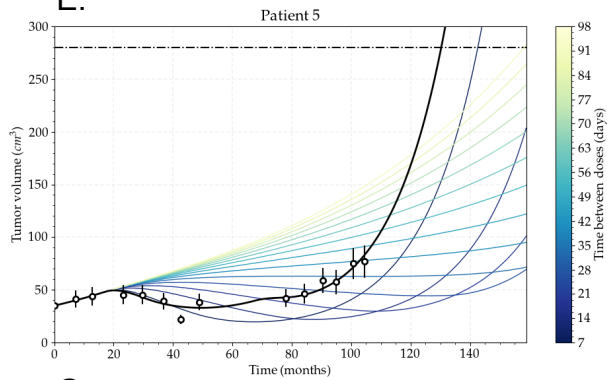

F.

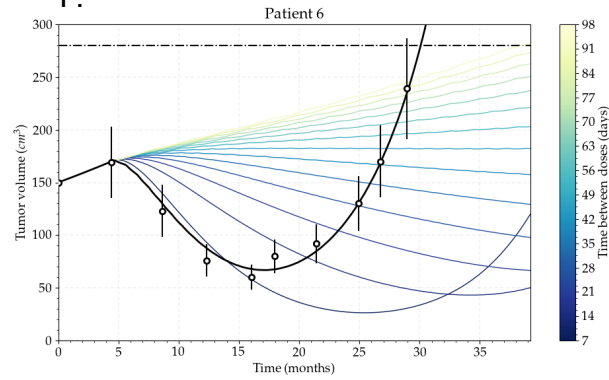

G.

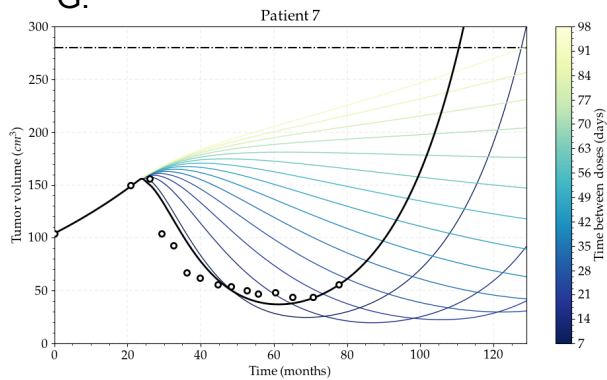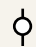

Data

Total tumor volume

Fatal tumor volume

Supplement: S3 Fig — Simulation of different experimental protocols consisting of spacing each single dose by a given number of days, from 7 to 98 (gradient lines). The longer the interval between doses, the longer the OS. However, tumor control is lost after a certain number of days between each dose, depending on each patient. Error bar represent 20% of error. (PDF) [file pcbi.1011208.s003.pdf]

**A.**

$$f(E) = \frac{1}{2} \left( 1 - \tanh \left( \frac{E - 0.05}{0.05} \right) \right)$$

C28 Vs ID21

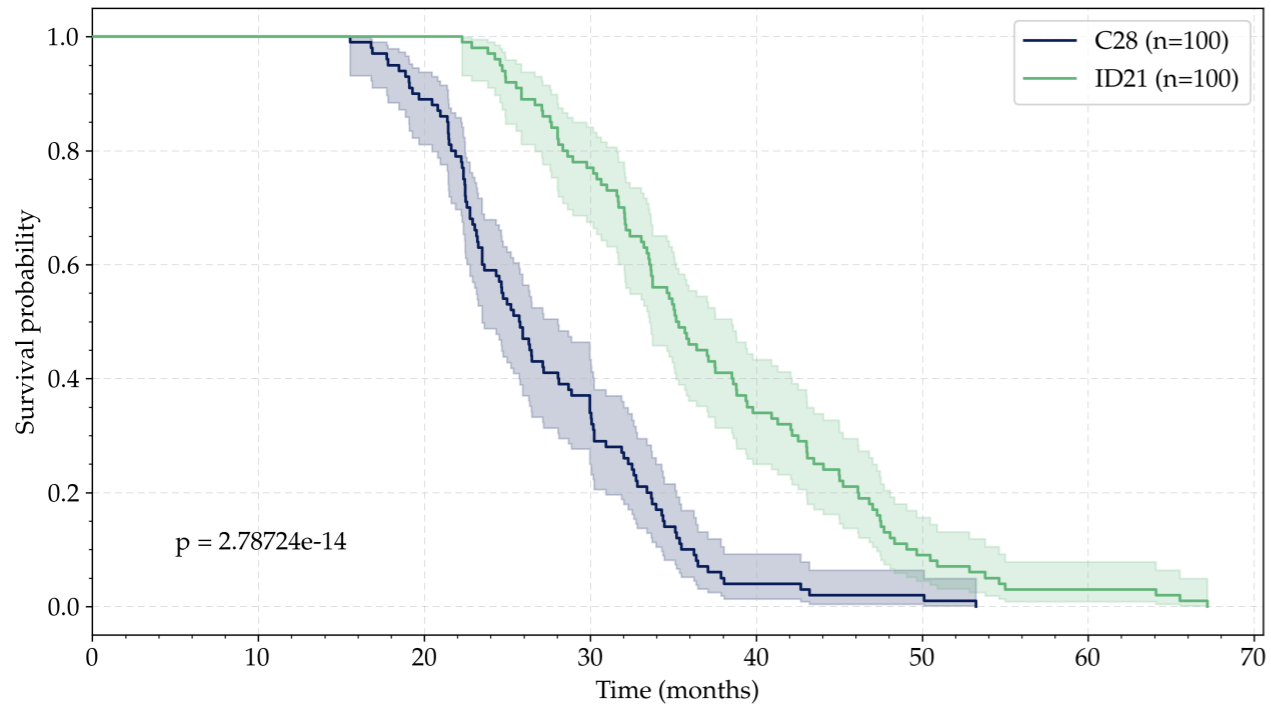**B.**

$$f(E) = \frac{1}{2} \left( 1 - \tanh \left( \frac{E - 0.1}{0.1} \right) \right)$$

C28 Vs ID21

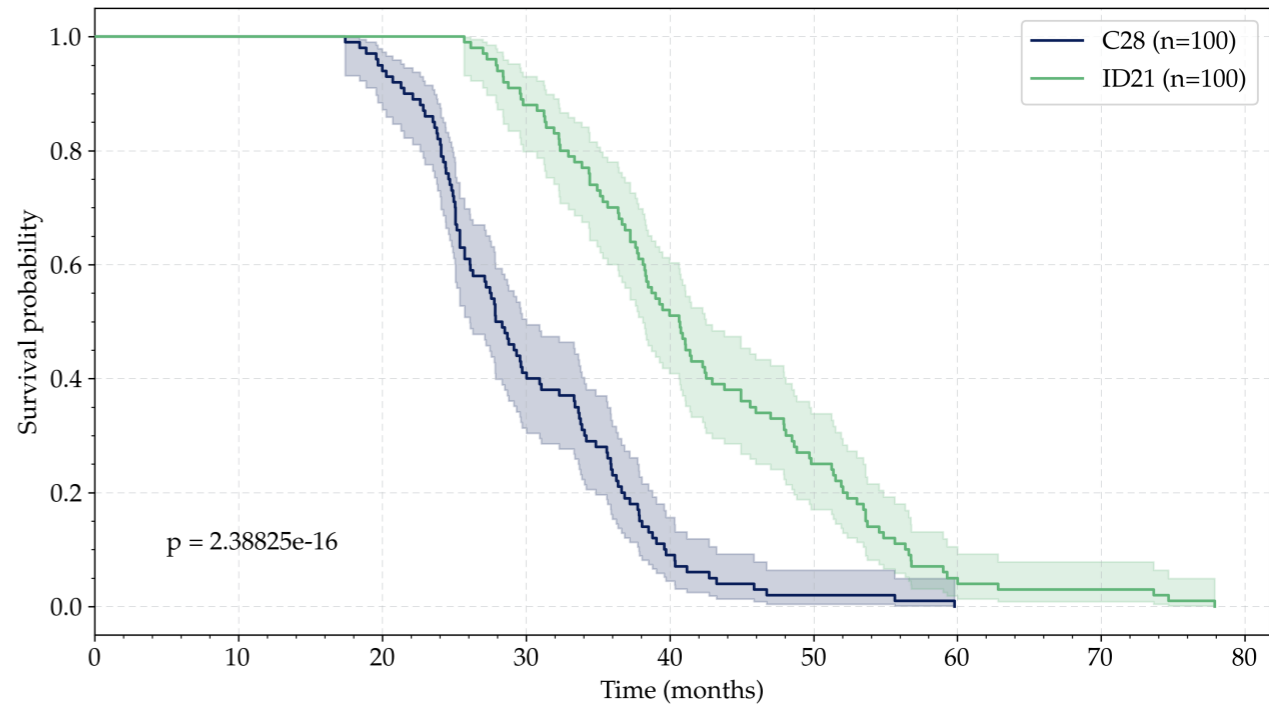

Supplement: S6 Fig — Results of clinical trials of the ID21 protocol against the C28 protocol with two different values of the fixed parameter that modifies the shape of the switching function between VPI and VP. Kaplan-Meier curves are shown with the results in terms of survival of these clinical trials. (A) f(E) = 1/2(1 − tanh((E − 0.05)/0.05)). (B) f(E) = 1/2(1 − tanh((E − 0.1)/0.1)). (PDF) [file pcbi.1011208.s006.pdf]

**A.**

C28 Vs ID14 (60 doses)

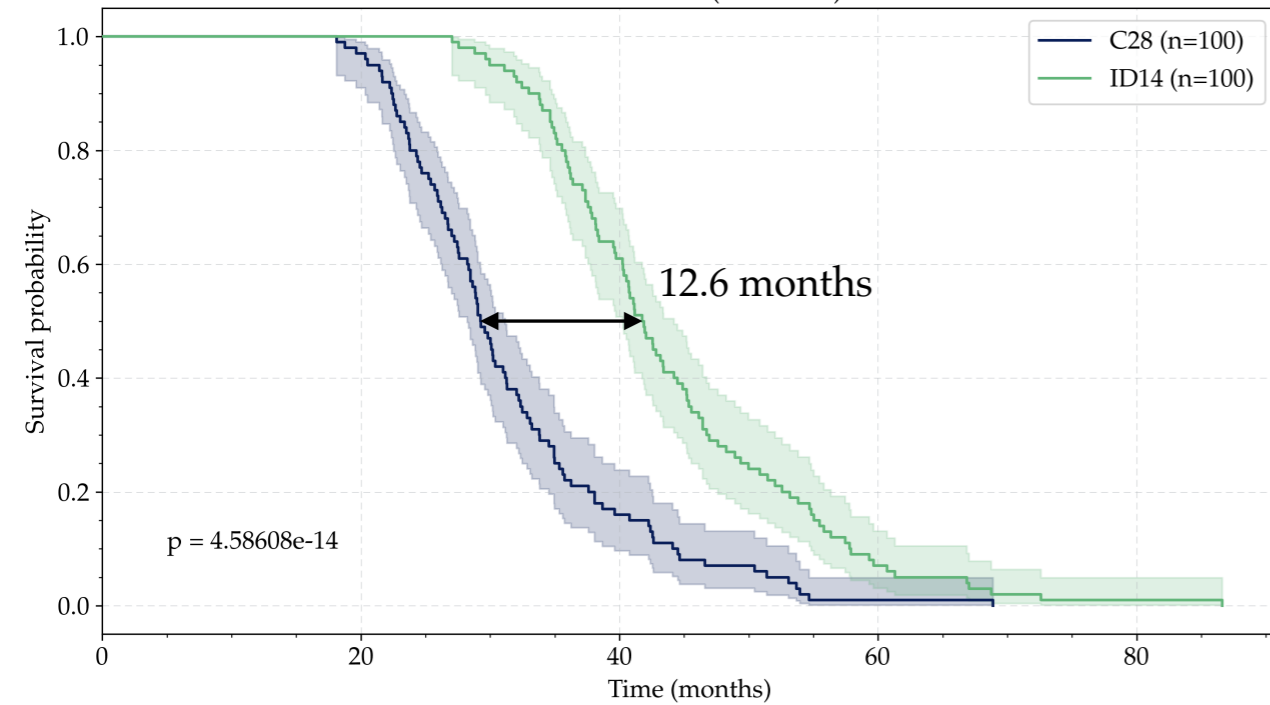**B.**

C28 Vs ID14 (120 doses)

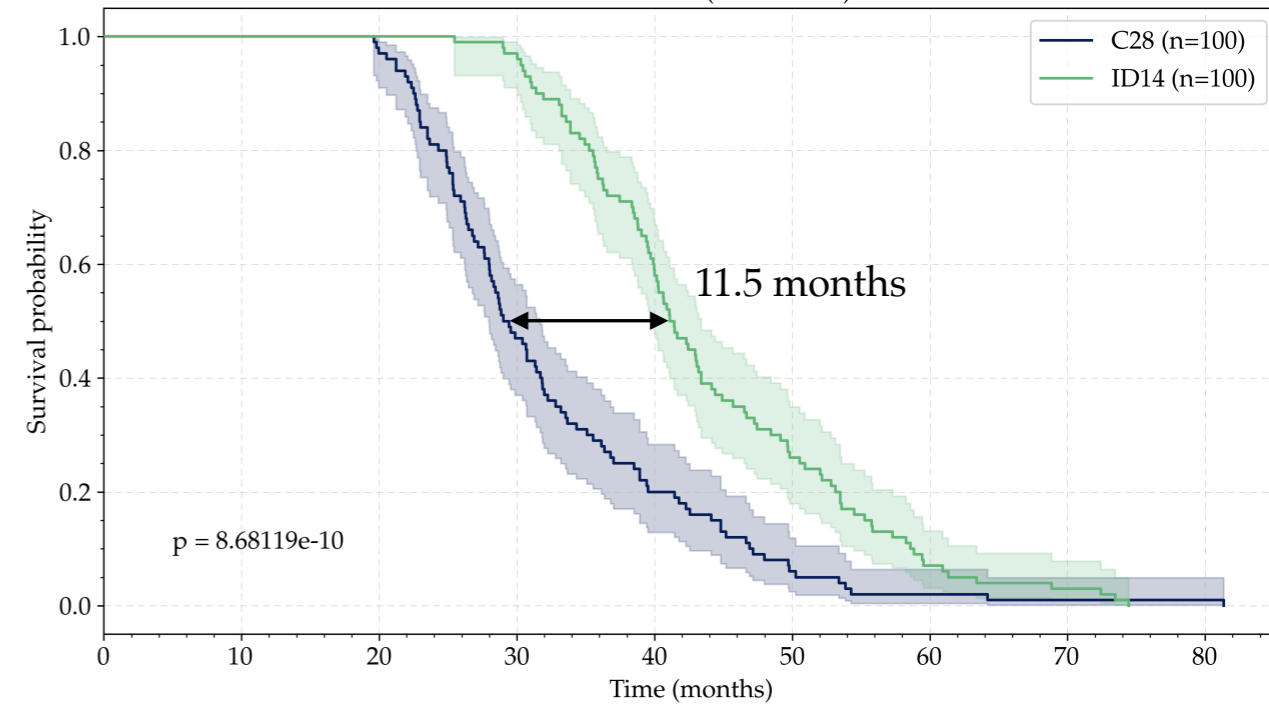**C.**

C28 Vs ID21 (60 doses)

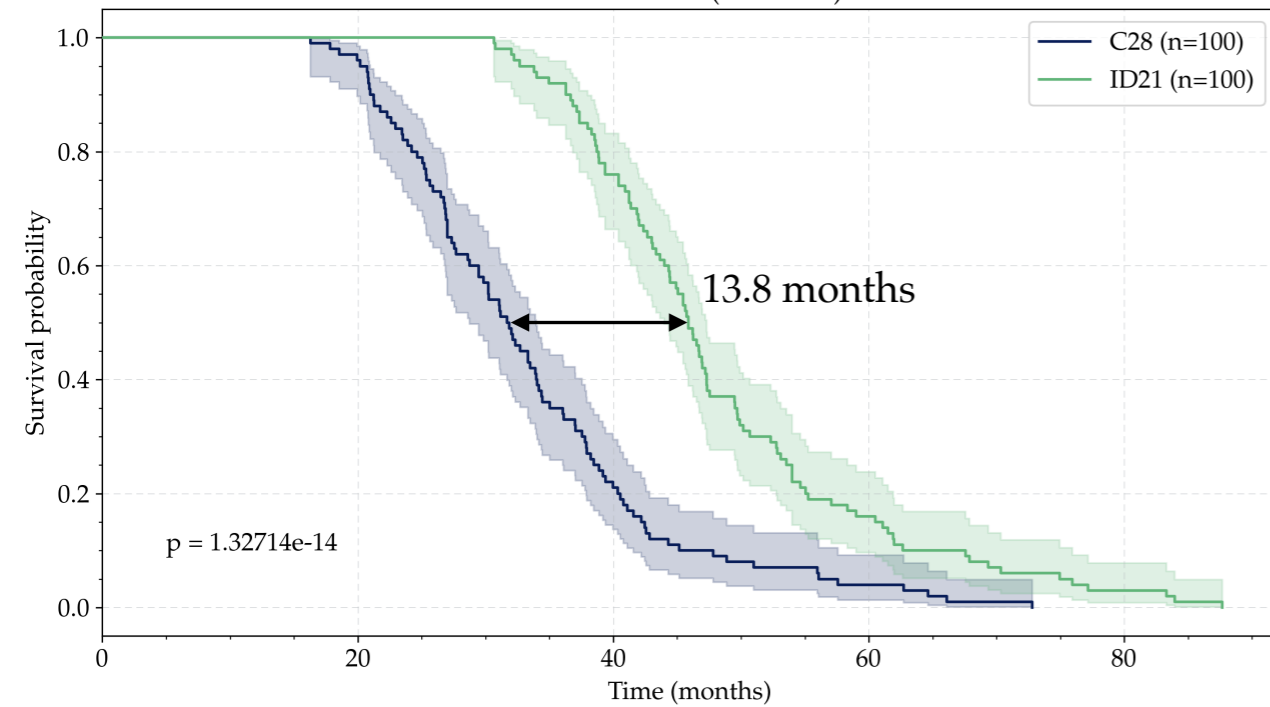**D.**

C28 Vs ID21 (120 doses)

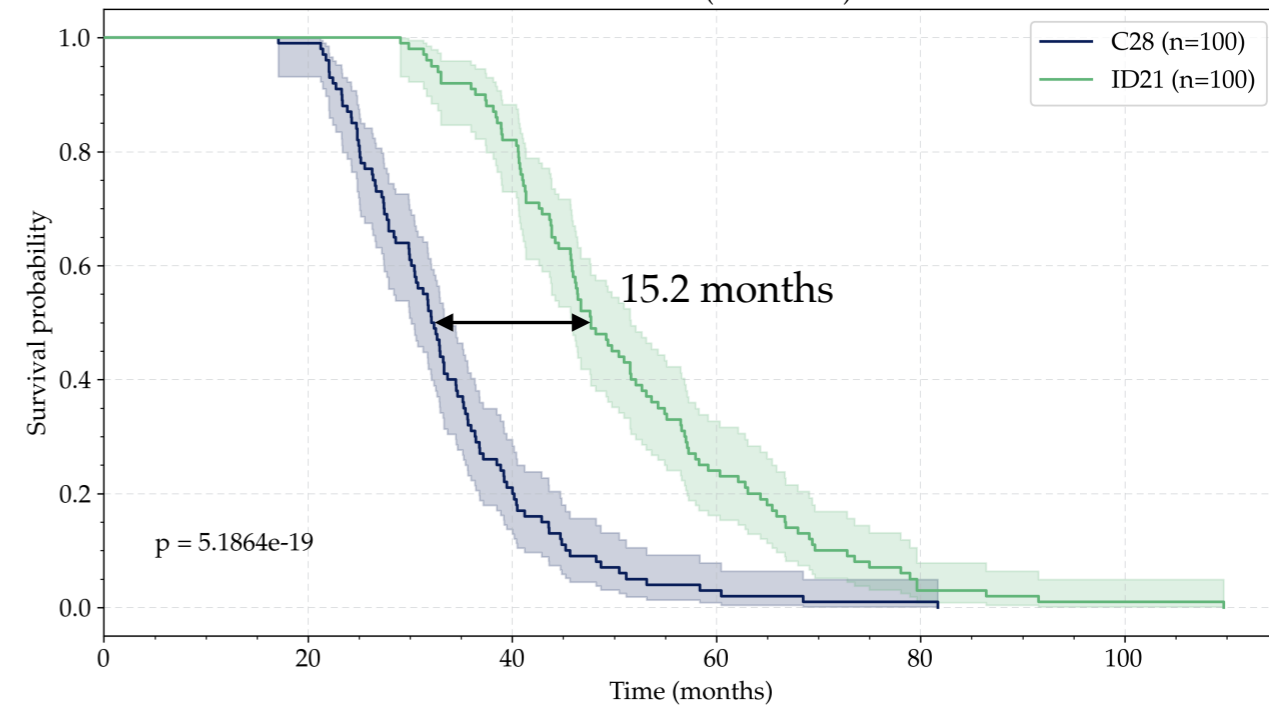

Supplement: S7 Fig — Kaplan-Meier curves showing the distribution of overall survival in virtual LGG patients undergoing TMZ treatment with ID14 and ID21 protocols (individual doses given every 14 or 21 days) against the classical C28 regime. Importantly, the number of drug cycles pre-set for all the virtual patients enrolled in the trial is the same. (A) Results for ID14 with 12 cycles. (B) Results for ID14 with 24 cycles. (C) Results for ID21 with 12 cycles. (D) Results for ID21 with 24 cycles. (PDF) [file pcbi.1011208.s007.pdf]

**A.**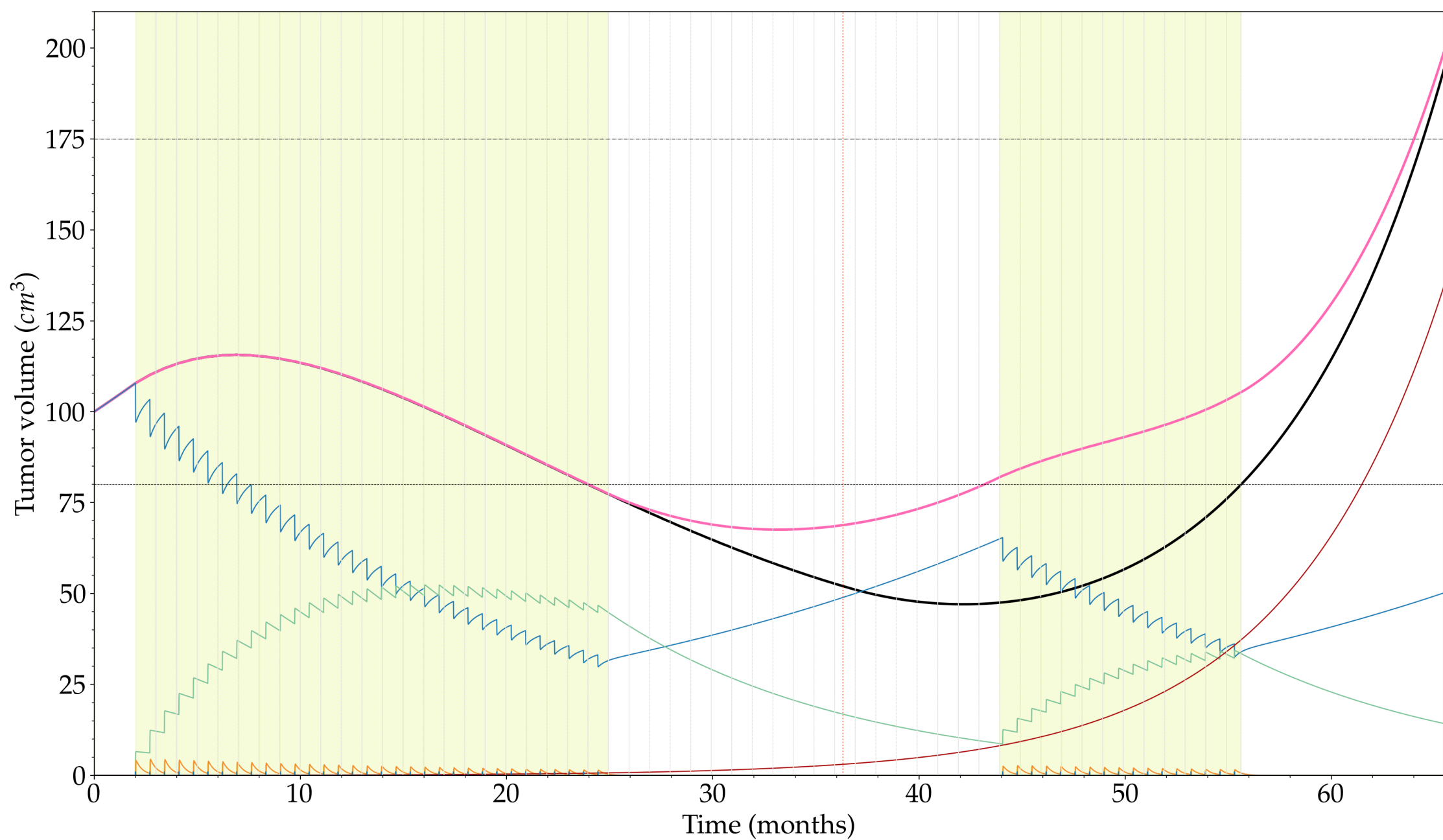**B.**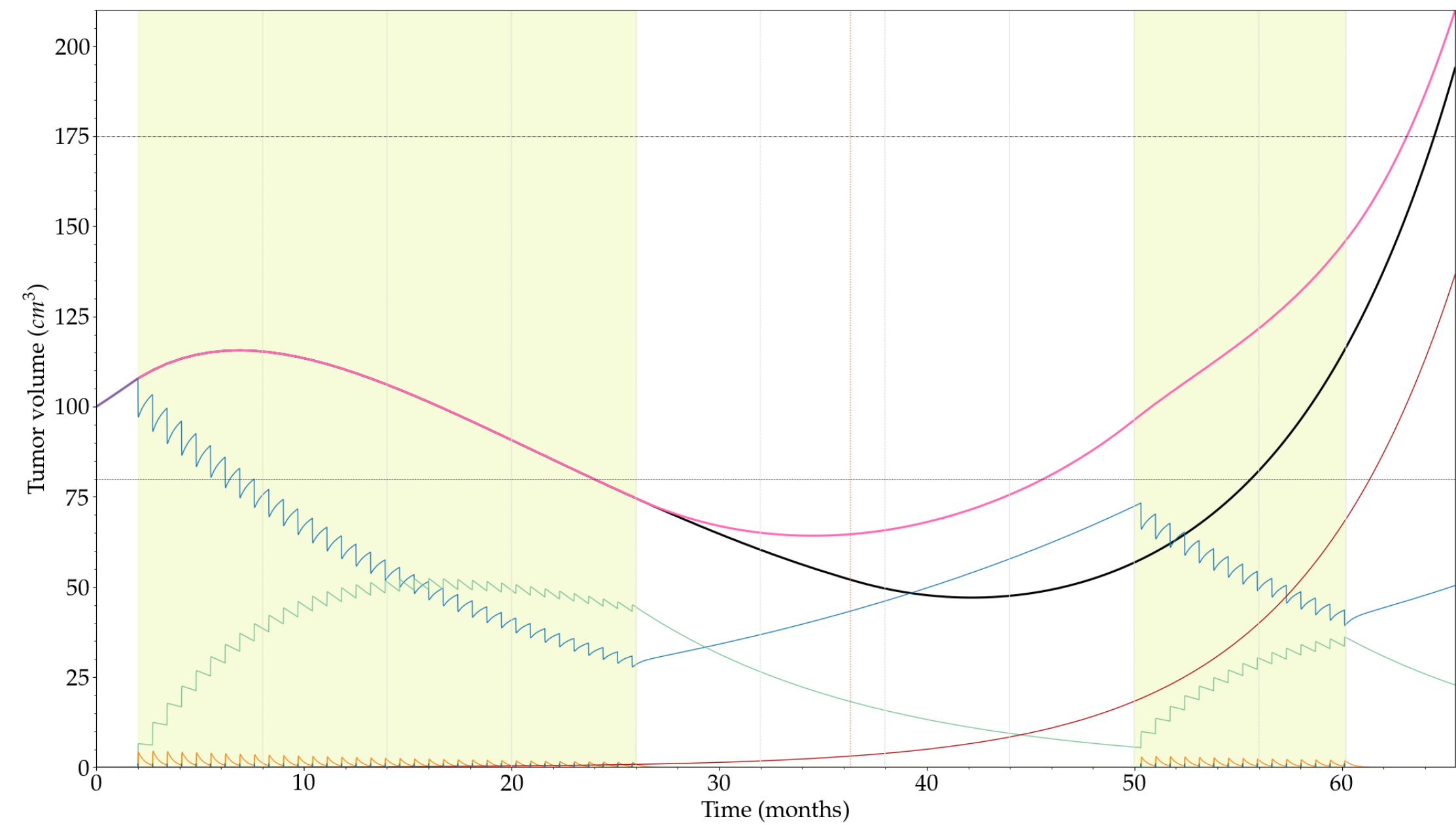

Supplement: S8 Fig — Time evolution of tumor volume for a virtual glioma patient undergoing TMZ under two different protocols. The black line corresponds to an ID21 protocol where 50 doses are equally spaced in time. The time at the end of the dose is indicated with a vertical dotted orange line. The pink line corresponds to the tumor volume with that same ID protocol but applying an adaptive variation. (A) The patient is screened every 30 days (gray dashed lines). (B) The patient is screened every 180 days (gray dashed lines). In both cases the doses are only applied during the next interval after the screenings if the tumor volume is higher than a certain proportion (taken as the 80% here) of the initial volume. The time periods when the doses are applied are indicated by a yellow background. (PDF) [file pcbi.1011208.s008.pdf]

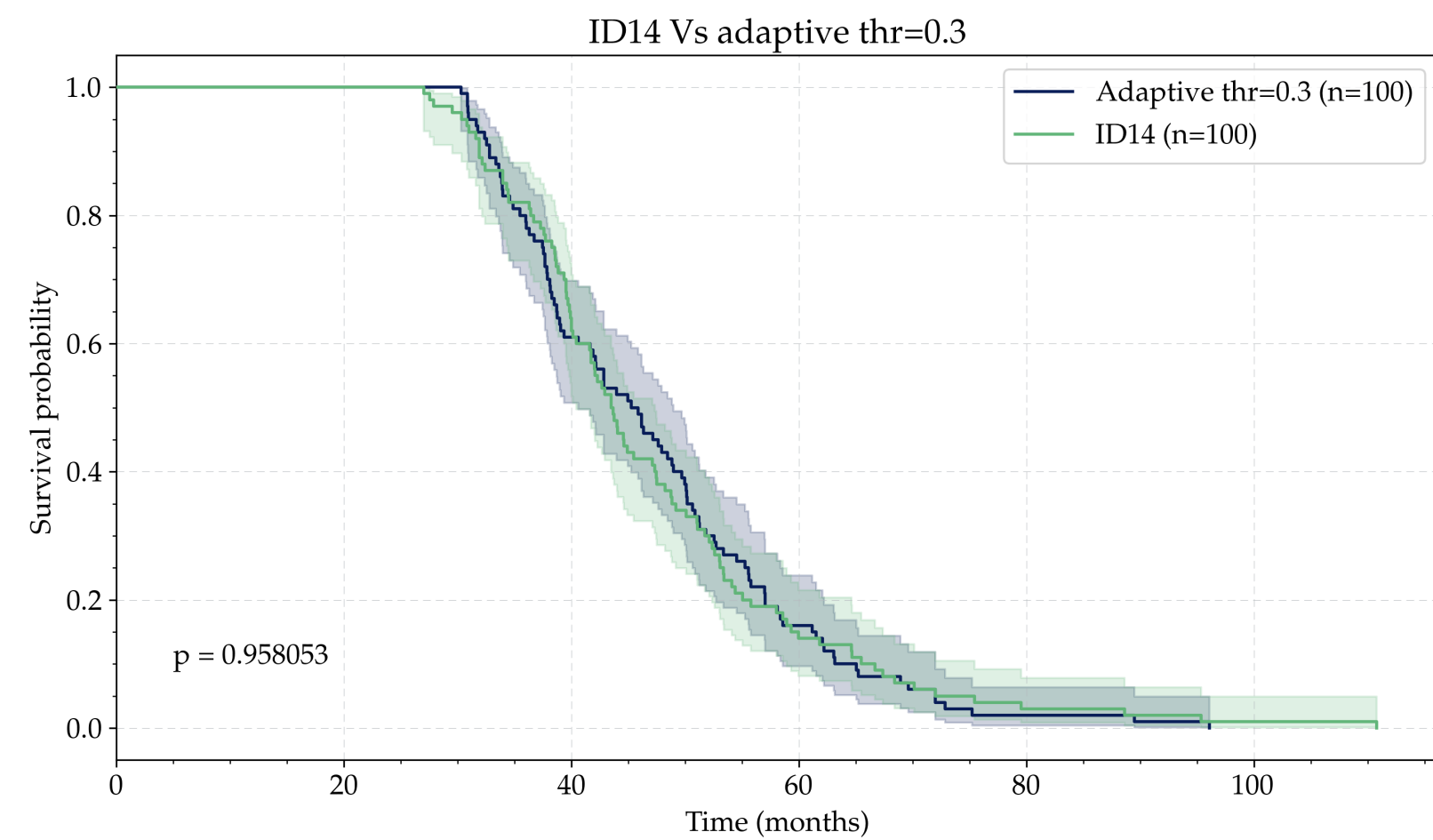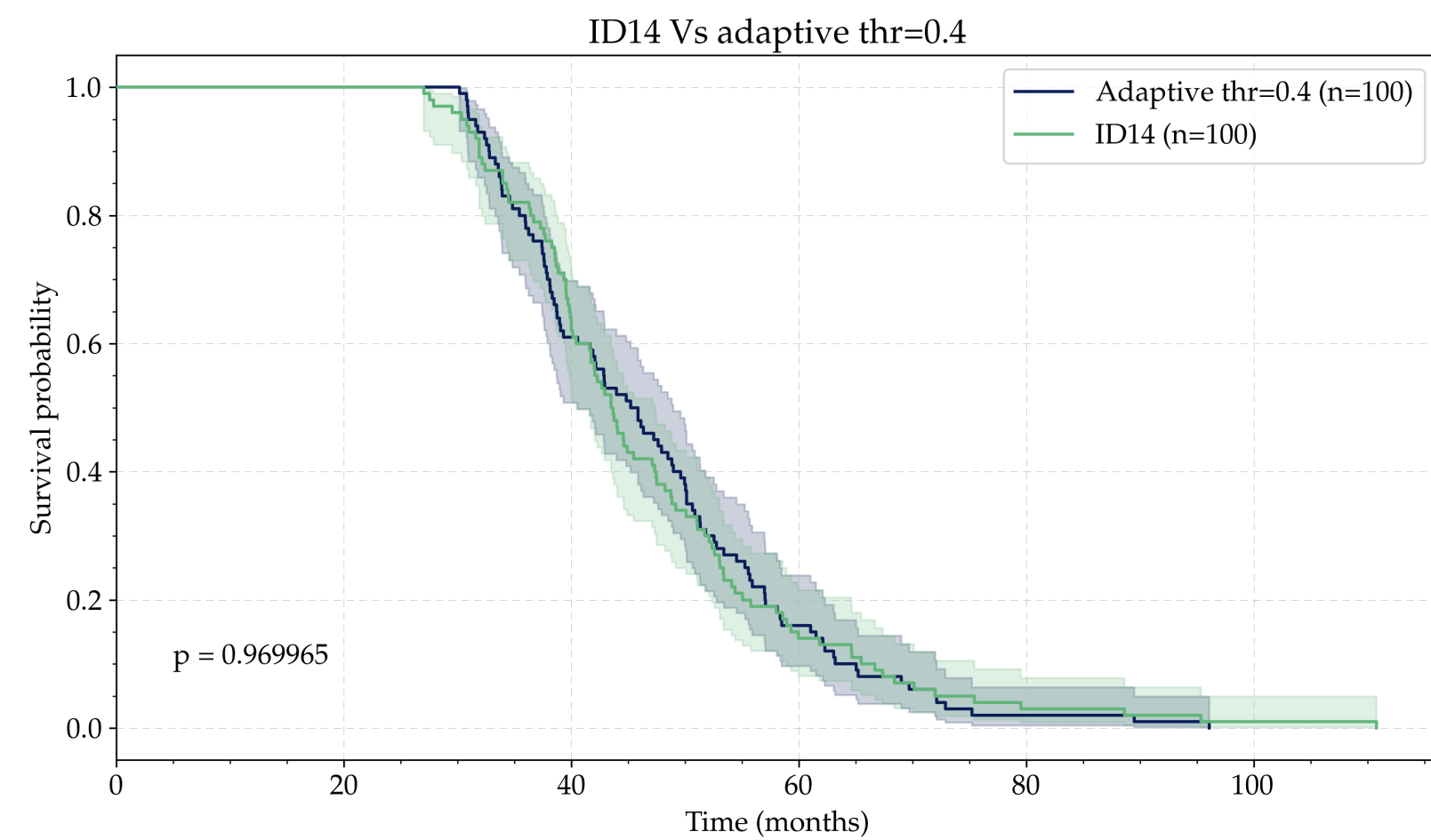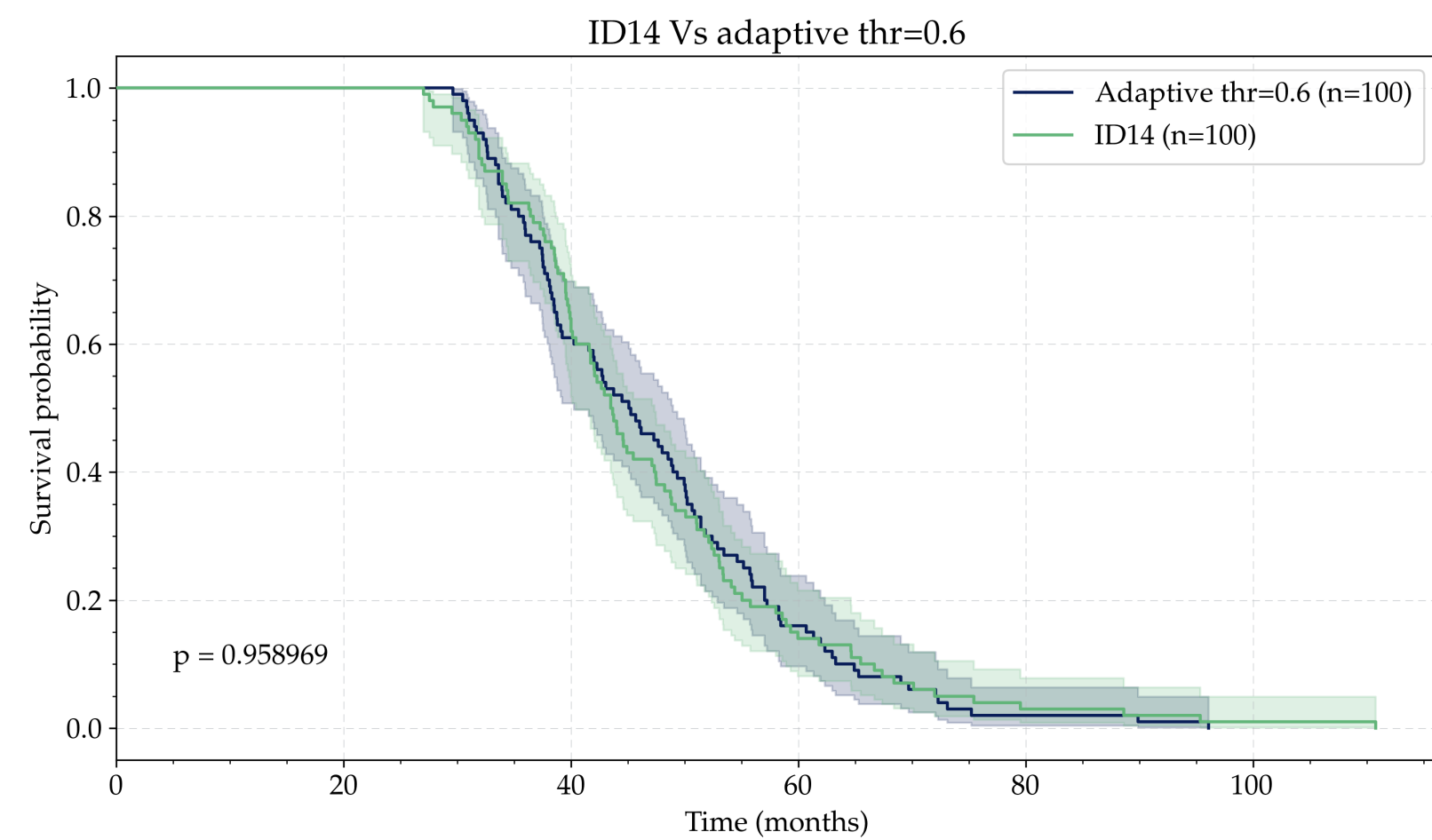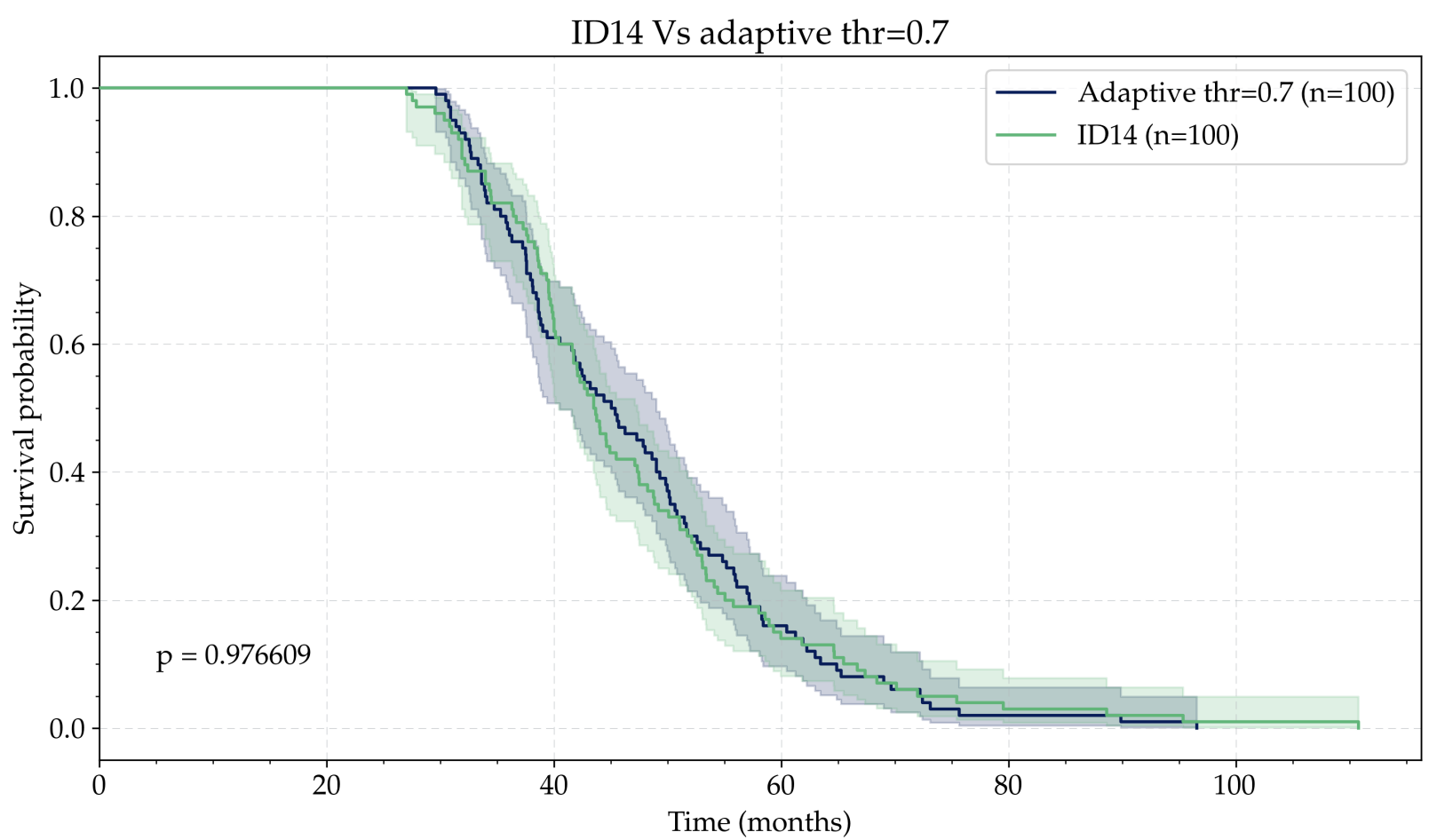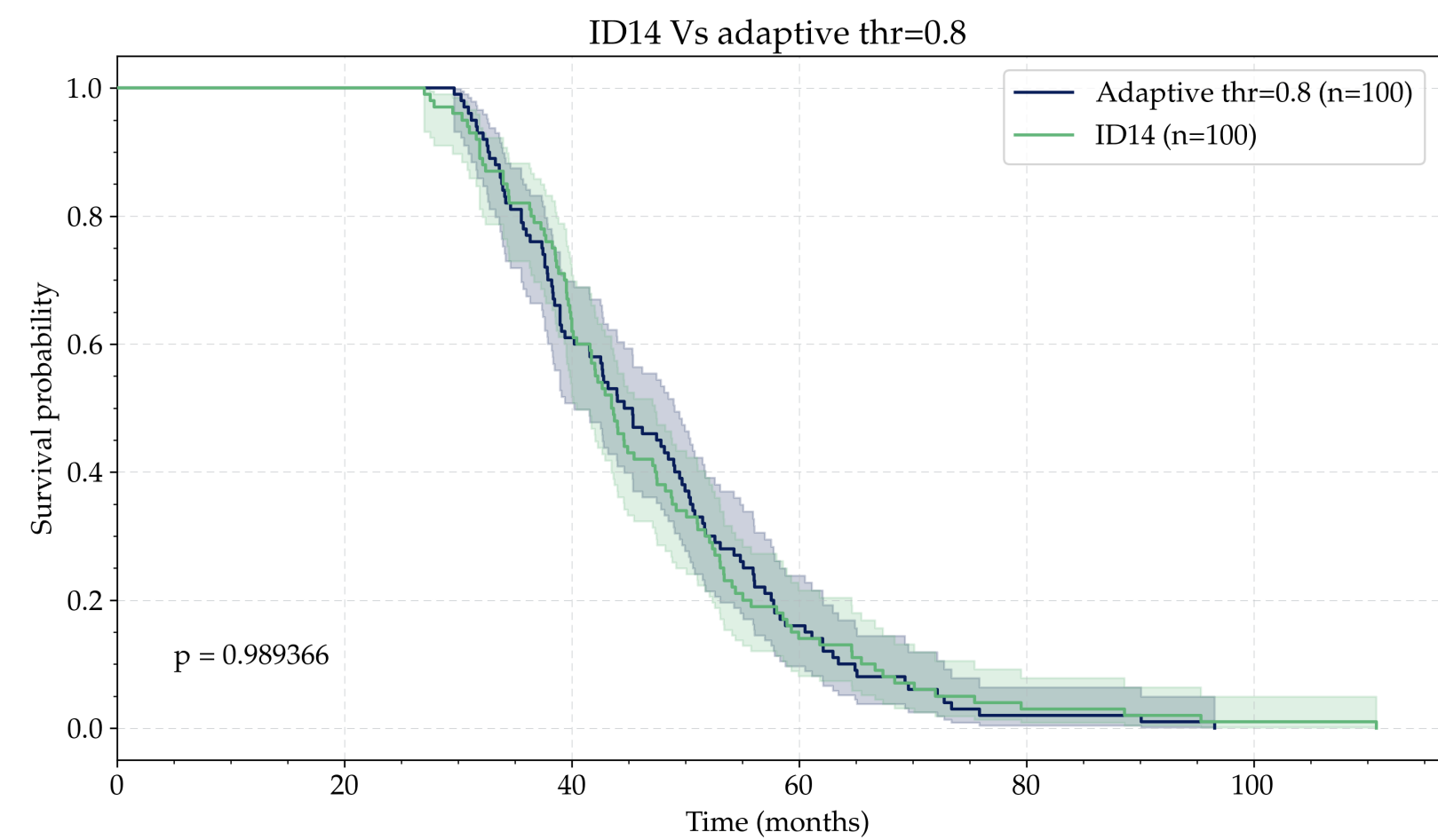

Supplement: S9 Fig — Kaplan-Meier curves showing the distribution of overall survival in virtual LGG patients undergoing TMZ treatment with ID14 protocols (individual doses given every 14 days) and their adaptive version (treatment interruption when the volume in 90-days periodic screenings is below a certain fraction of the volume at diagnosis). Six different trials were simulated for different values of the threshold deciding the administration of the treatment between 30% and 80%. The trials showed no significant difference between the survival of patients with the ID protocol or its adaptive version. (PDF) [file pcbi.1011208.s009.pdf]

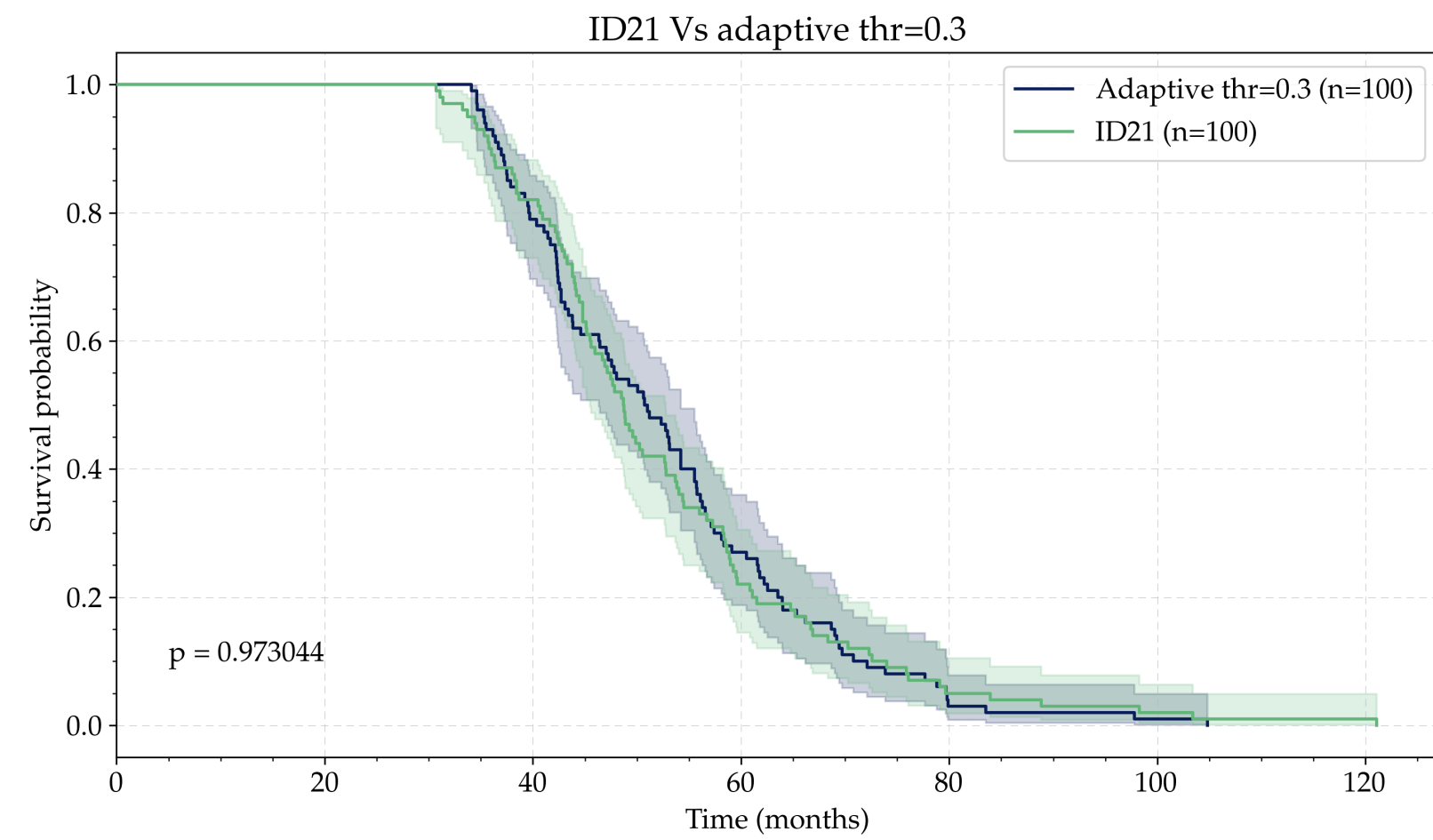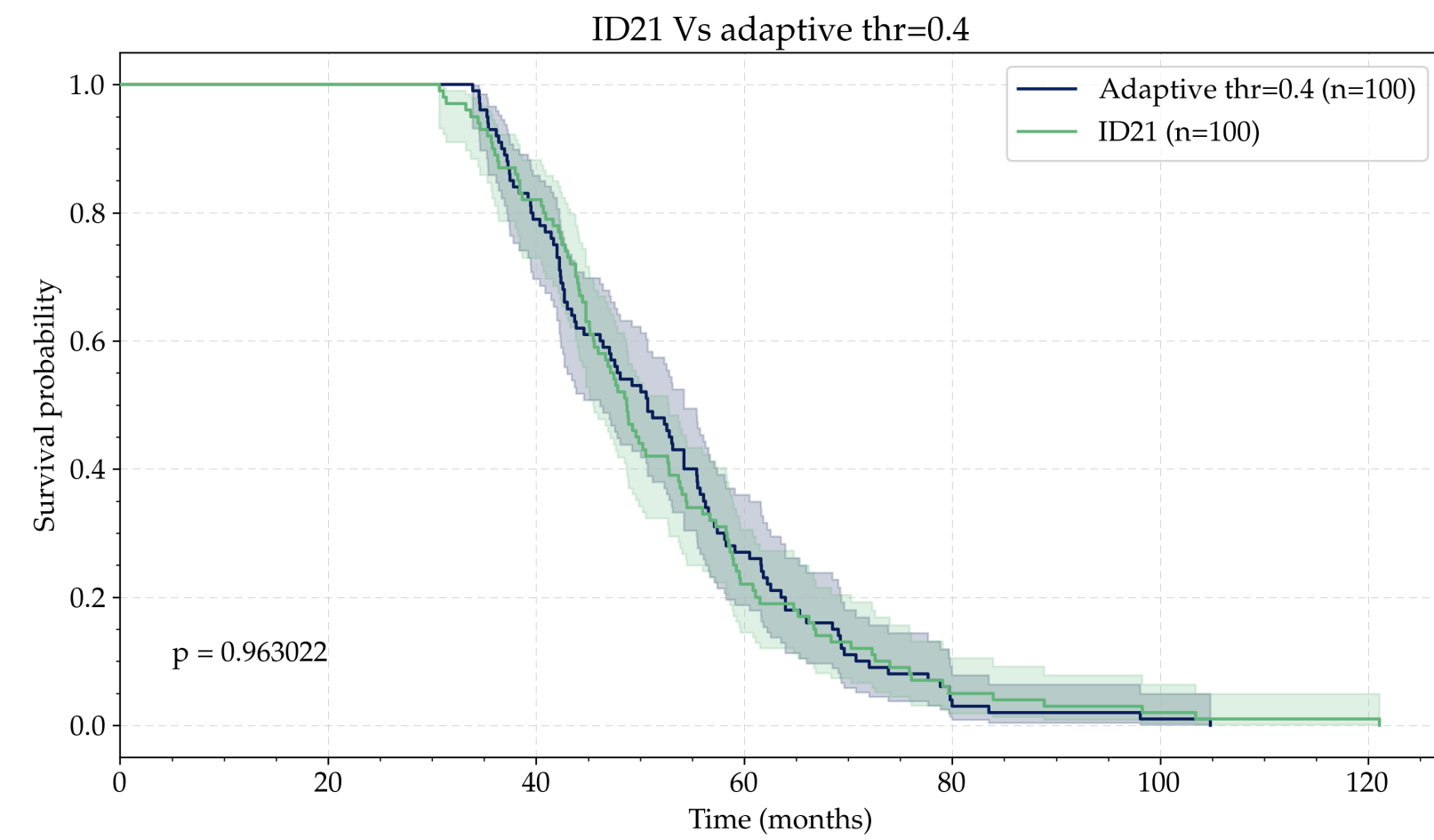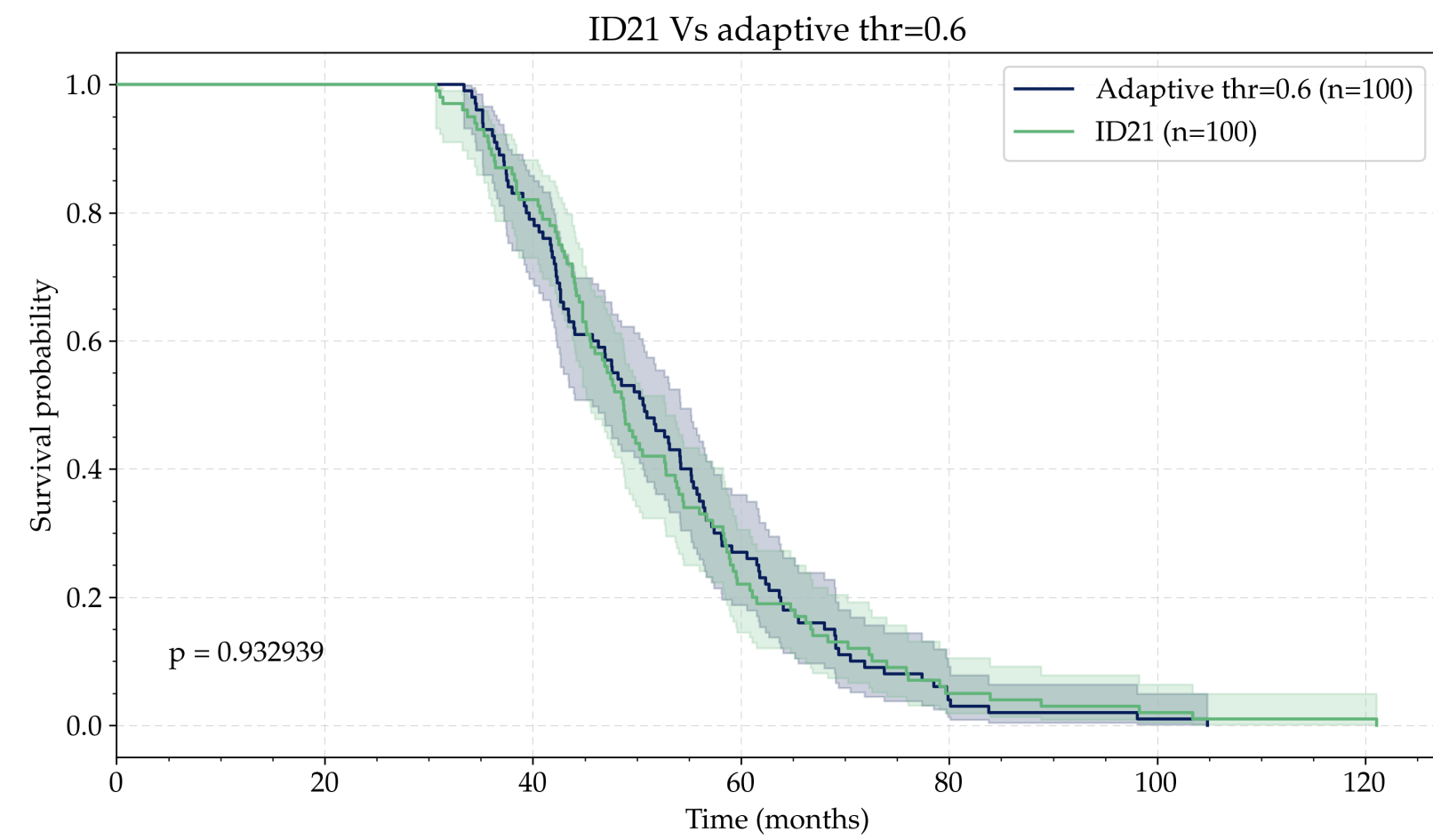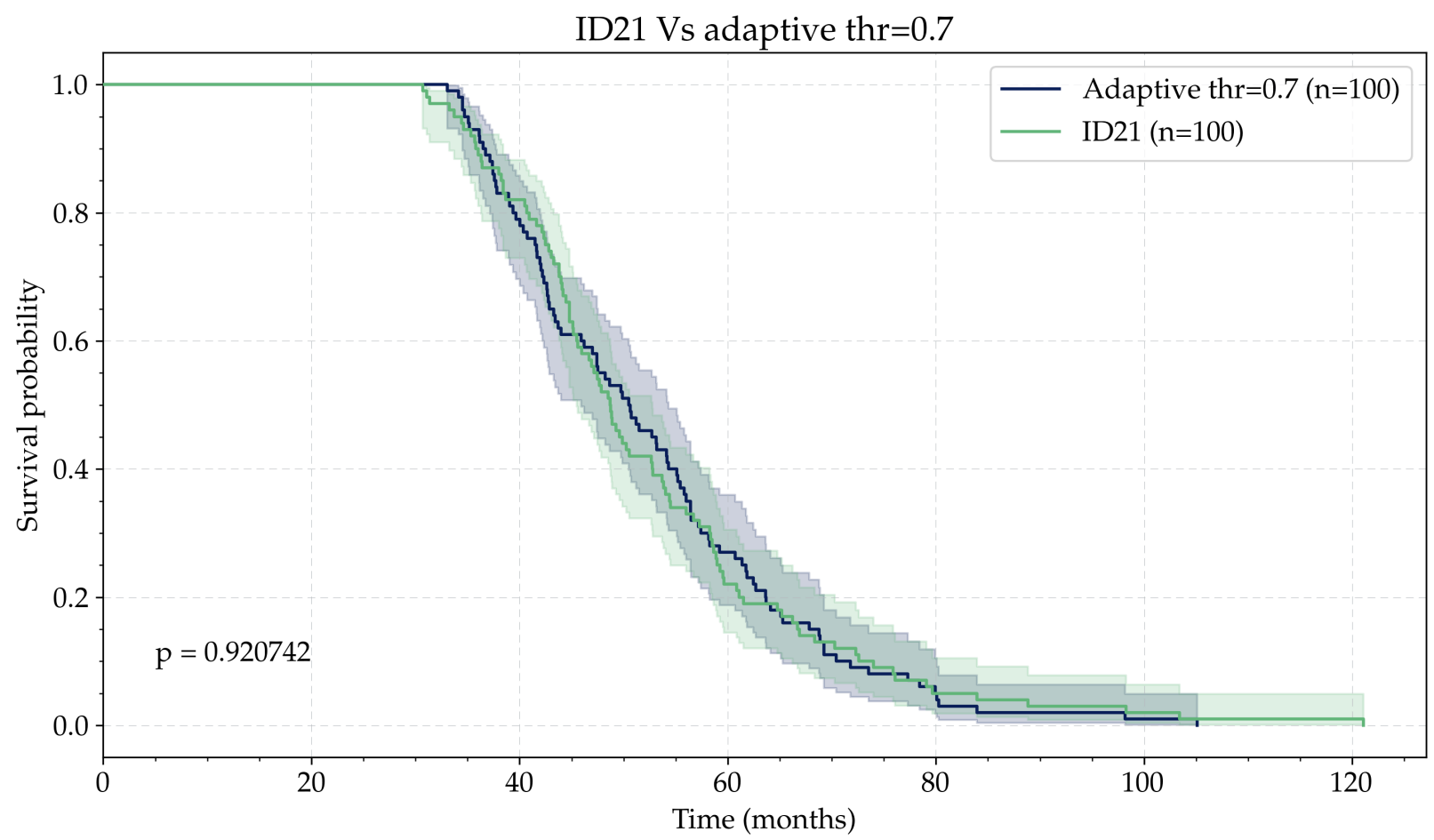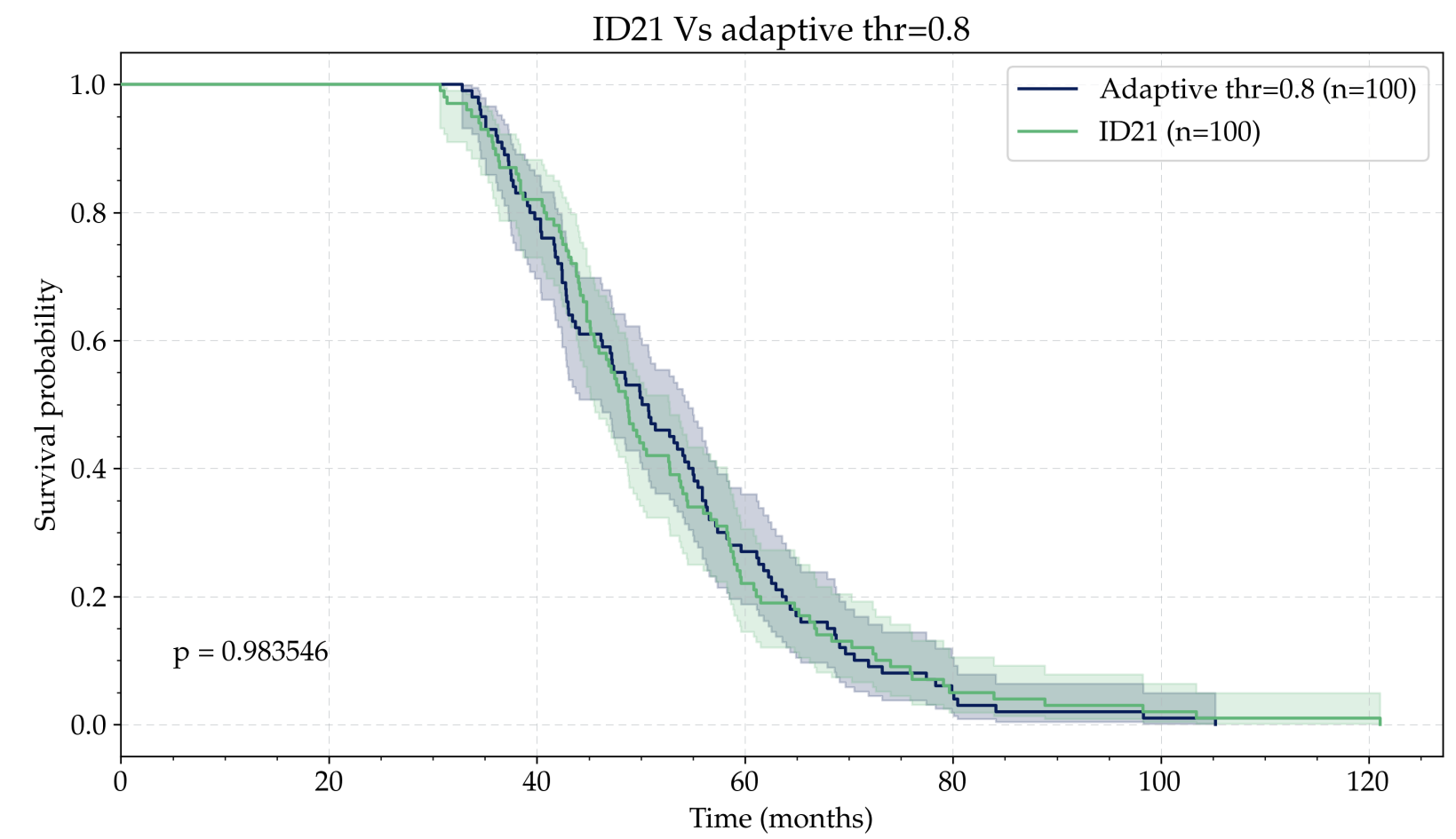

Supplement: S10 Fig — Kaplan-Meier curves showing the distribution of overall survival in virtual LGG patients undergoing TMZ treatment with ID14 protocols (individual doses given every 21 days) and their adaptive version (treatment interruption when the volume in 90-days periodic screenings is below a certain fraction of the volume at diagnosis). Six different trials were simulated for different values of the threshold deciding the administration of the treatment between 30% and 80%. The trials showed no significant difference between the survival of patients with the ID protocol or its adaptive version. (PDF) [file pcbi.1011208.s010.pdf]

## Example 2

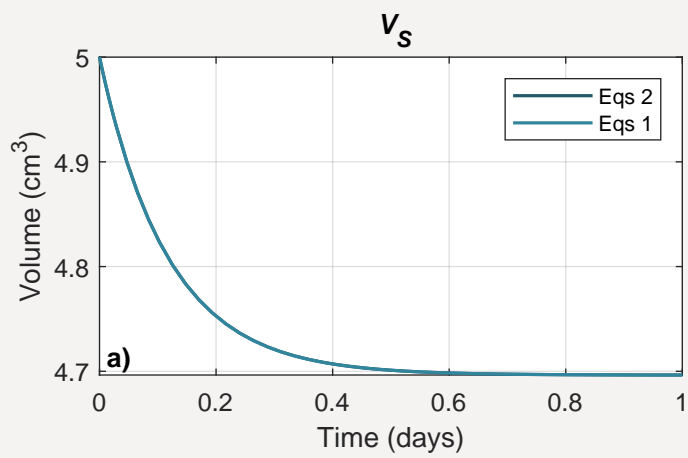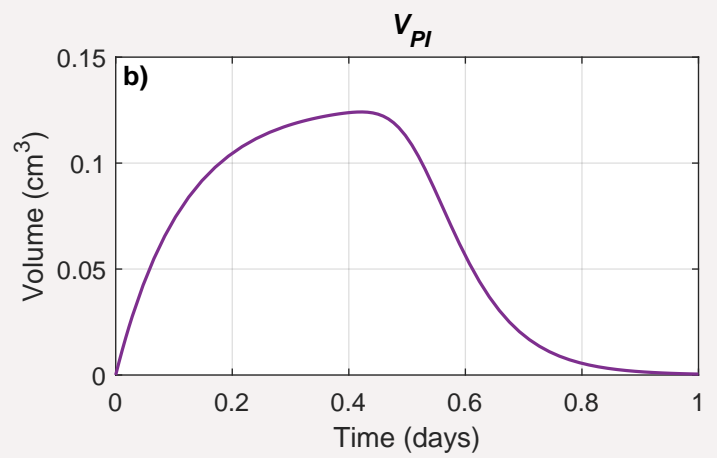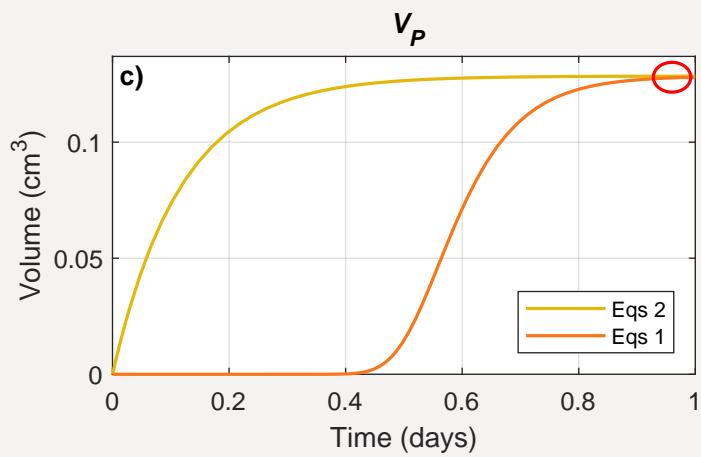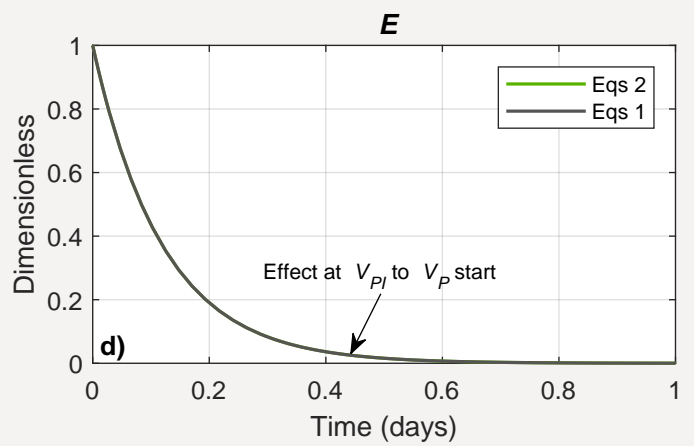

Supplement: S2 File — How it was determined. (ZIP) [file pcbi.1011208.s012.zip › S2_File.zip/Example2.pdf]

## Example 1

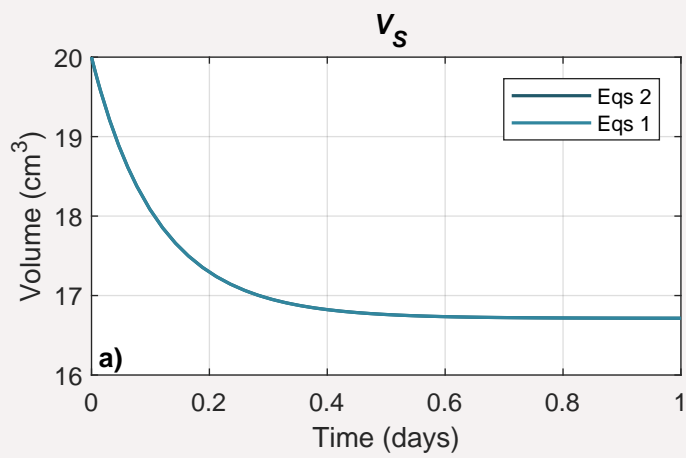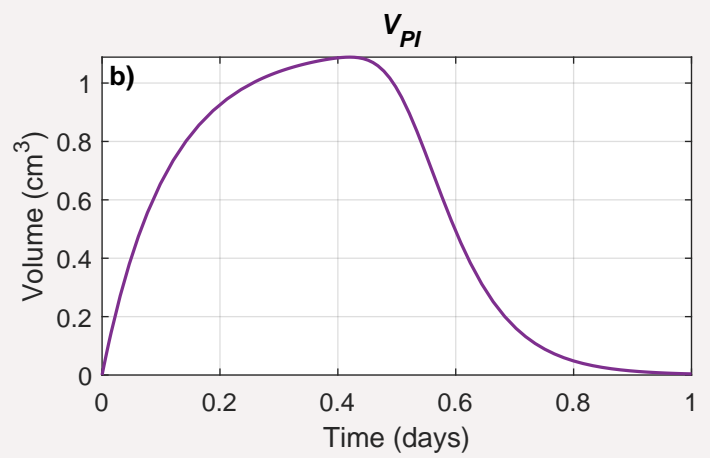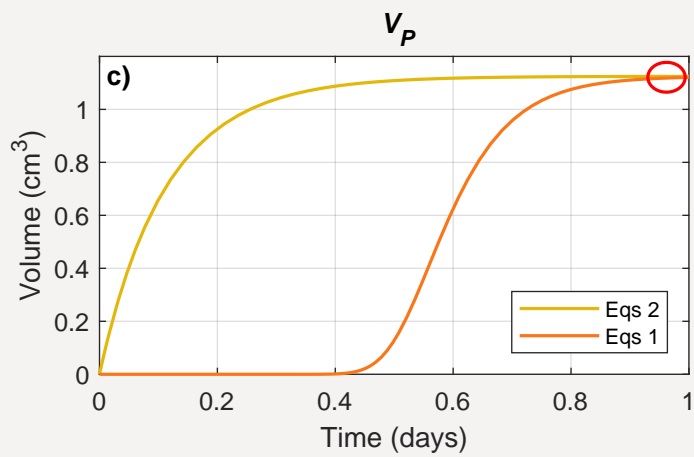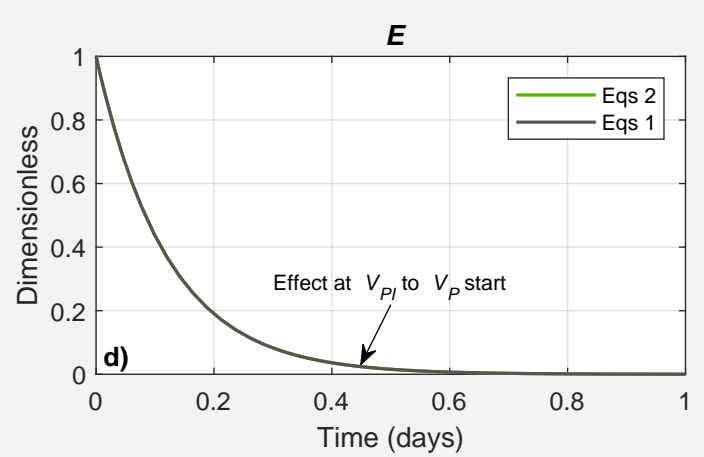

Supplement: S2 File — How it was determined. (ZIP) [file pcbi.1011208.s012.zip › S2_File.zip/Example1.pdf]
