# Supplementary material for: Overcoming chemotherapy resistance in low-grade gliomas: A computational approach
Source: PLoS Comput Biol. 2023 Nov 20;19(11):e1011208. doi: 10.1371/journal.pcbi.1011208 (PMC10695391; doi:10.1371/journal.pcbi.1011208)
Supplement: S5 Fig — Results of clinical trials of the ID21 protocol against the C28 protocol with four different values of the fixed parameter β. Kaplan-Meier curves are shown with the results in terms of survival of these clinical trials. (A) β = 2 × 10^−2^ day−1. (B) β = 5 × 10^−2^ day−1. (C) β = 5 × 10^−1^ day−1. (D) β = 1 day−1. (PDF) [file pcbi.1011208.s005.pdf]

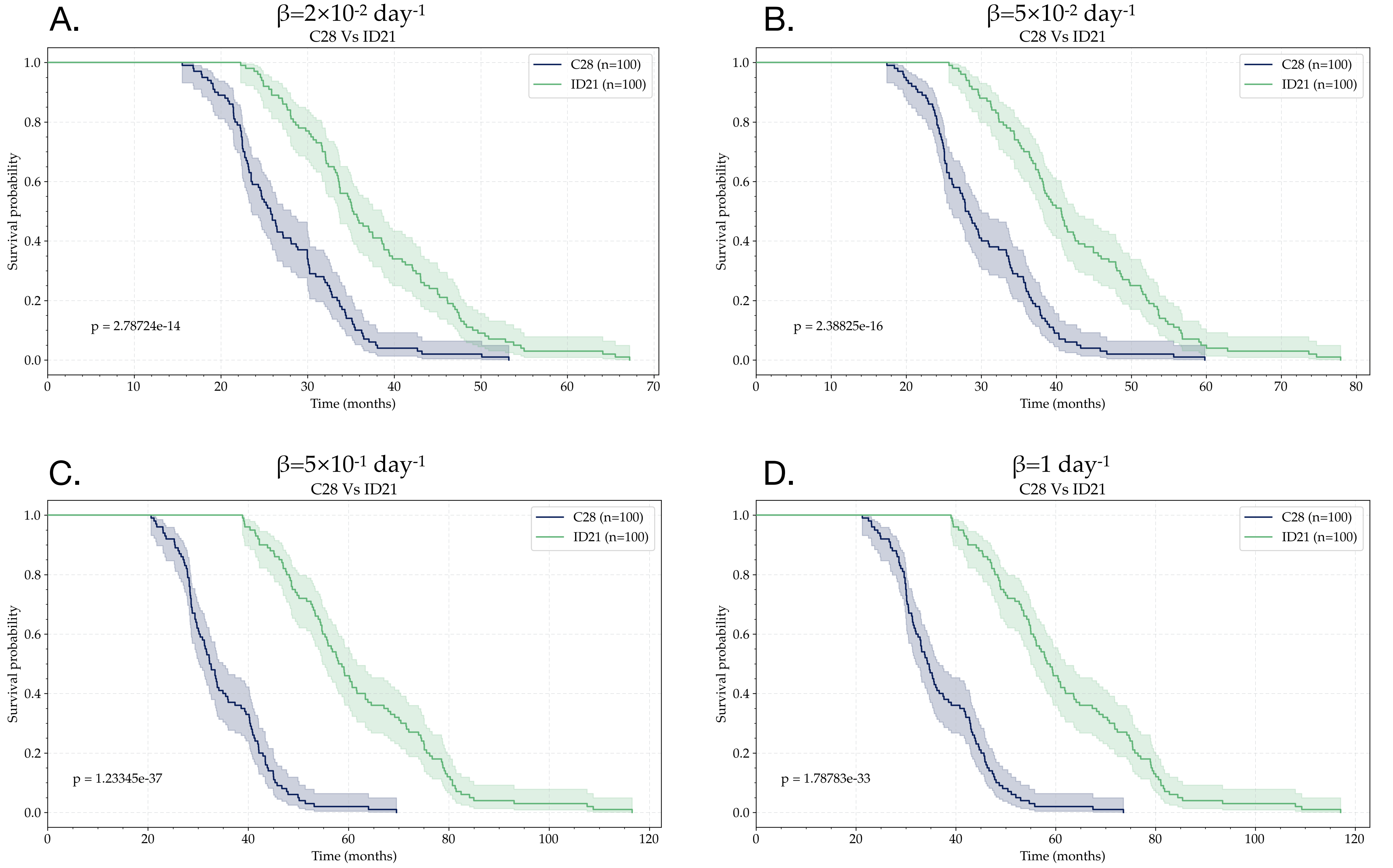
A.
β=2×10-2 day-1
C28 Vs ID21
C28 (n=100)
ID21 (n=100)
p = 2.78724e-14
Survival probability
Time (months)
B.
β=5×10-2 day-1
C28 Vs ID21
C28 (n=100)
ID21 (n=100)
p = 2.38825e-16
Survival probability
Time (months)
C.
β=5×10-1 day-1
C28 Vs ID21
C28 (n=100)
ID21 (n=100)
p = 1.23345e-37
Survival probability
Time (months)
D.
β=1 day-1
C28 Vs ID21
C28 (n=100)
ID21 (n=100)
p = 1.78783e-33
Survival probability
Time (months)
