# Supplementary material for: Overcoming chemotherapy resistance in low-grade gliomas: A computational approach
Source: PLoS Comput Biol. 2023 Nov 20;19(11):e1011208. doi: 10.1371/journal.pcbi.1011208 (PMC10695391; doi:10.1371/journal.pcbi.1011208)
Supplement: S2 File — How it was determined. (ZIP) [file pcbi.1011208.s012.zip › S2_File.zip/S2 File.pdf]

Explanation of the activation function $f(E)$

As stated in the "Formulation of the mathematical model" paper section, a temporary compartment V_{PI} was added in order to prevent one single TMZ dose from producing fully resistant cell volume V_R. This can happen due to iterative nature of typical numerical methods used to approximate the solution of ordinary differential equations. The desired biological behavior that we sought to describe is a two steps process in which a number of sensitive cells first transform to a reversible persister state under the effect of TMZ and then, if the exposure is repeated, transit to their fully resistant state. Consequently, the same dose application cannot trigger the same cell to transform to a persister and a resistant state. This forces us to inclue an intermediate persister population and an activation function $f(E)$ with the following characteristics:

1. **High concentrations of the drug E should not allow the transit from V_{PI} to V_P, i.e. there is a delay from the time of application of the drug to the moment in which cells fix their persister phenotype.** Sensitive cells cannot be transformed in resistant in a significative number with the application of a single TMZ dose. Due to the continuous approach of our model it is not possible to completely avoid that a single TMZ dose generates fully resistant cells, but this behavior has to be prevented. As patients received more than one dose, however, small deviations can be tolerated as the resistant cells appearing cannot be imputed to an individual application of the drug.

2. **The transition time to the fully persister phenotype V_P must be lower than one day.** According to the usual TMZ application consisting of pills with a minimum uptake period of one day, at the time of application of a subsequent TMZ dose, the formed persisters should be already free to go back to sensitive or well transit to resistant as a function of the new applied dose concentration, that is, they should belong to the V_P compartment. Therefore, cells in the temporary V_{PI} compartment have a time of one day to pass to the persister compartment V_P so the transition to a fully resistant state can happen in the minimum spacing considered between doses. In other words, all the sensitive cells that become persister by a specific TMZ dose must be in the persister compartment V_P one day after TMZ administration. Otherwise, cells that are supposed to be persister would not be affected by the next TMZ dose due to they are still in the V_{PI} compartment.

With this considerations into account, $f(E)$ was selected by solving simplified versions of our model and adapt their behavior to the previous biological rules. As the drug concentration E is eliminated very quickly in comparison to the tumor time evolution, a good approximation of the cell population dynamics, while the drug is present can be obtained by neglecting the terms that do not contain the effect E. Additionally, the term $-\alpha_2 V_P E$ is neglected in the simplified models so the exact amount of persister cells produced by one TMZ dose can be tracked. This way we arrive to the models

$$\frac{dV_S}{dt} = -\psi V_S E - \alpha_1 V_S E \tag{1a}$$

$$\frac{dV_{PI}}{dt} = \alpha_1 V_S E - V_{PI} f(E) \tag{1b}$$

$$\frac{dV_P}{dt} = V_{PI} f(E) \tag{1c}$$

$$\frac{dE}{dt} = -\lambda E \tag{1d}$$

$$\frac{dV_S}{dt} = -\psi V_S E - \alpha_1 V_S E \tag{2a}$$

$$\frac{dV_P}{dt} = \alpha_1 V_S E \tag{2b}$$

$$\frac{dE}{dt} = -\lambda E \tag{2c}$$

which are solved together with the initial conditions $V_S(0) = V_{S0}$, $V_{PI}(0) = V_P(0) = 0$ and $E(0) = 1$. Specific values were assigned to the parameters and to V_{S0}. The form of $f(E)$ that made system 1 (Eqs 1) meet the two previous criteria was chosen. System 2 (Eqs 2) was used to verifies criterion 2. The selected function was:

$$f(E) = 7.5\left(1 - \tanh\left(\frac{E-0.01}{0.01}\right)\right). \tag{3}$$

This function satisfies both criteria regardless of parameters values and V_{S0} (see Fig 1 and 2).

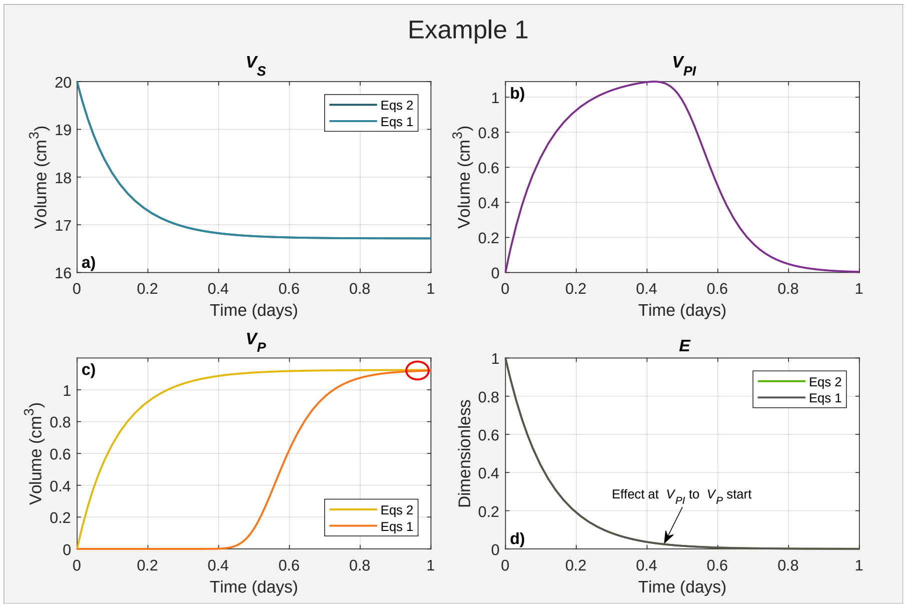

Fig 1. The $f(E)$ selected satisfies the imposed biological criteria. (a, c, d) Evolution of sensitive volume, persister volume and the effect, respectively, according to both systems of equations. b) Dynamic of V_{PI} volume. The fulfillment of criteria 1 is indicated with the black arrow in subplot d) and the fulfillment of criteria 2 with the red ellipse in subplot c). The curves in a) and d) overlap because cell dynamics are the same in both systems.$V_{S0} = 20$ cm^3, $\psi = 0.98$ cm^3/day, $\alpha_1 = 0.51$ cm^3/day and $\lambda = 8.3$ day^{-1}.

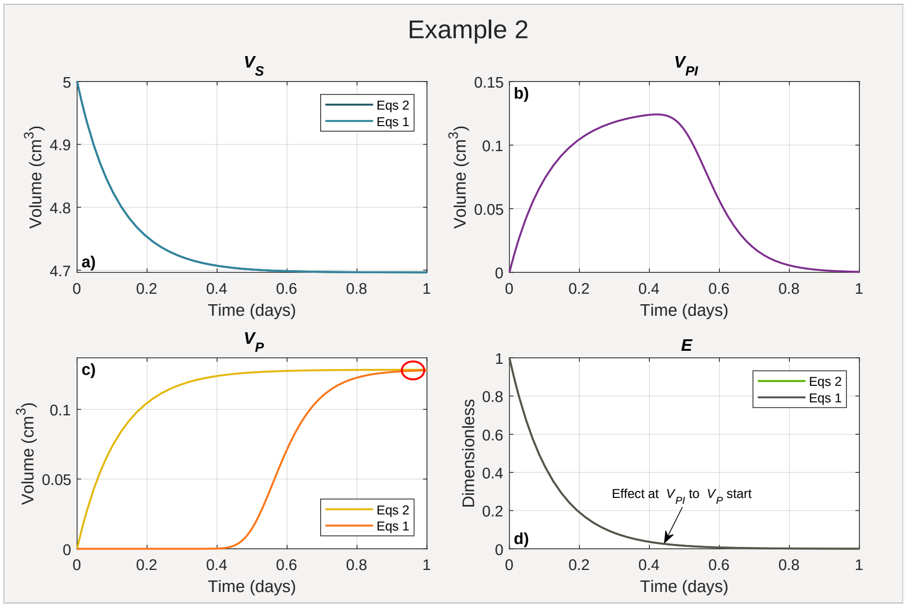

Fig 2. The $f(E)$ selected satisfies the imposed biological criteria. (a, c, d) Evolution of sensitive volume, persister volume and the effect, respectively, according to both systems of equations. b) Dynamic of V_{PI} volume. The fulfillment of criteria 1 is indicated with the black arrow in subplot d) and the fulfillment of criteria 2 with the red ellipse in subplot c). The curves in a) and d) overlap because cell dynamics are the same in both systems.$V_{S0} = 5$ cm^3, $\psi = 0.3$ cm^3/day, $\alpha_1 = 0.22$ cm^3/day and $\lambda = 8.3$ day^{-1}.
