# Supplementary material for: Overcoming chemotherapy resistance in low-grade gliomas: A computational approach
Source: PLoS Comput Biol. 2023 Nov 20;19(11):e1011208. doi: 10.1371/journal.pcbi.1011208 (PMC10695391; doi:10.1371/journal.pcbi.1011208)
Supplement: S3 File — In later fits, the value β was fixed due to the proximity of this parameter to β = 0.1 day−1 in all the studied cases. This file shows the results of these preliminary fits and the values obtained for the parameters. (PDF) [file pcbi.1011208.s013.pdf]

S3 File. Preliminary fits

Patient 1

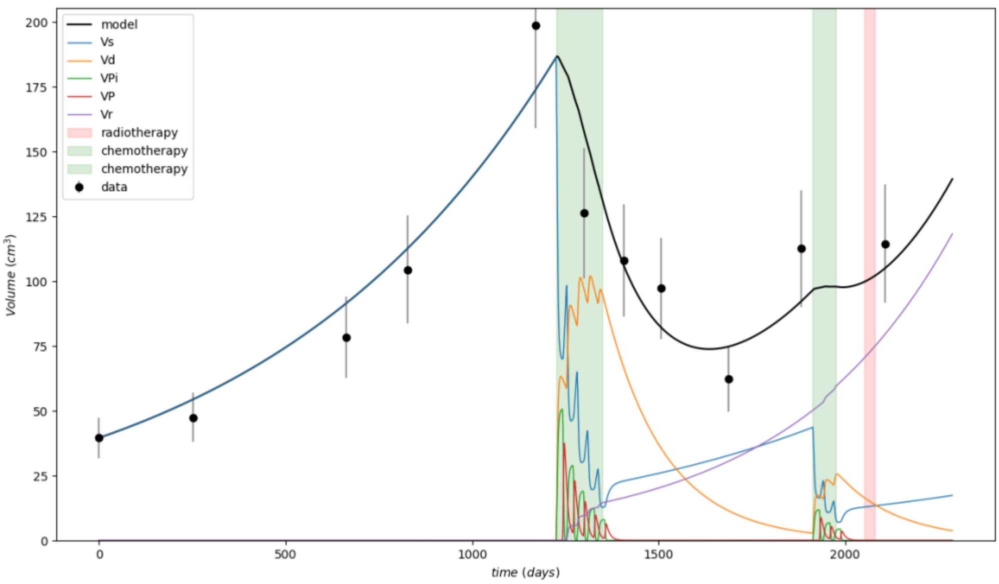

ρ_1	ρ_2	ρ_3	α_2	α_3	α_4	β	λ_1
0.000127	0.0022	0.0062	0.039	0.03	0.03	0.1	1.5

Patient 2

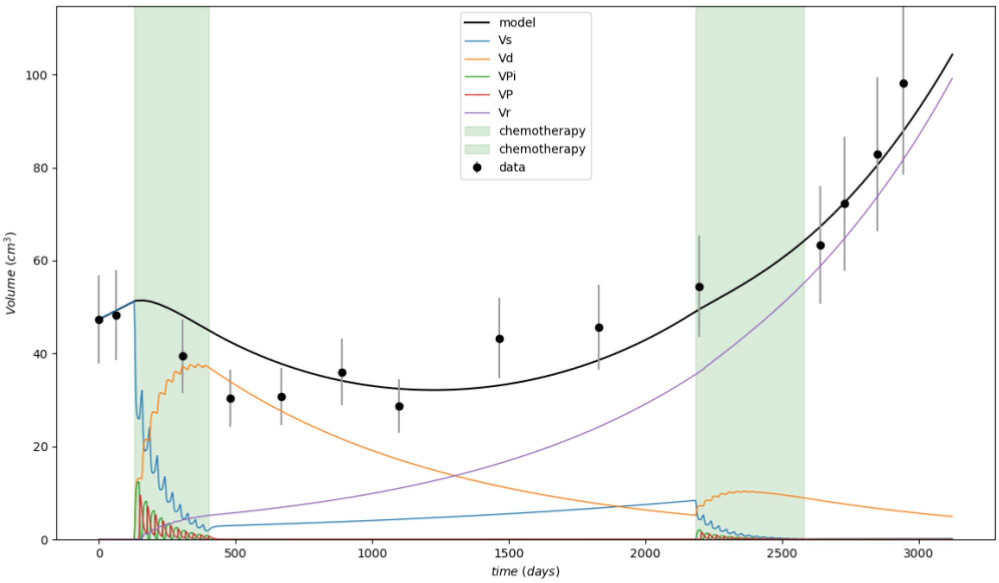

ρ_1	ρ_2	ρ_3	α_2	α_3	α_4	β	λ_1
0.0006	0.00105	0.00111	0.0257	0.0227	0.0203	0.087	1.5

Patient 3

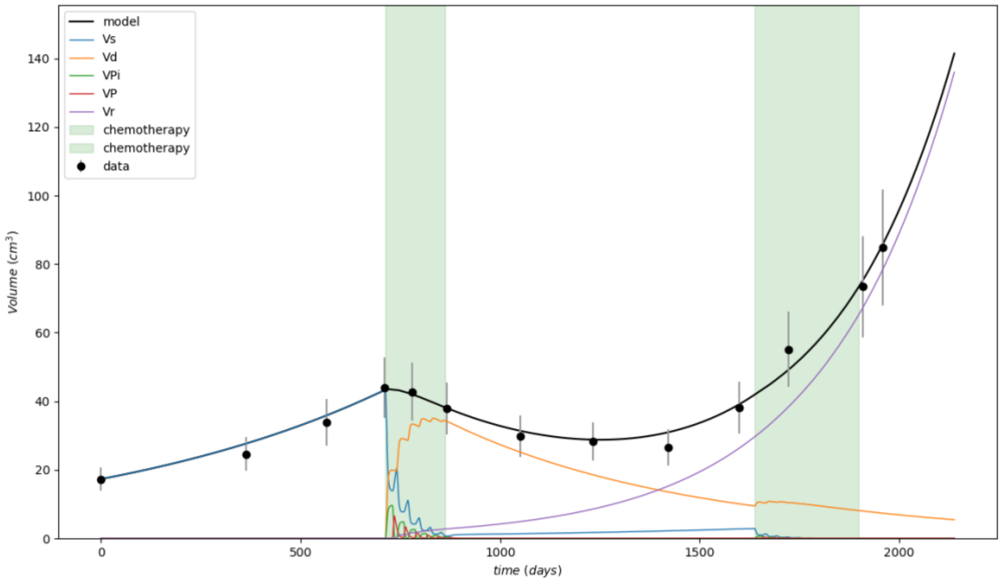

ρ_1	ρ_2	ρ_3	α_2	α_3	α_4	β	λ_1
0.000129	0.00307	0.00167	0.055	0.026	0.05	0.14	1.5

Patient 4

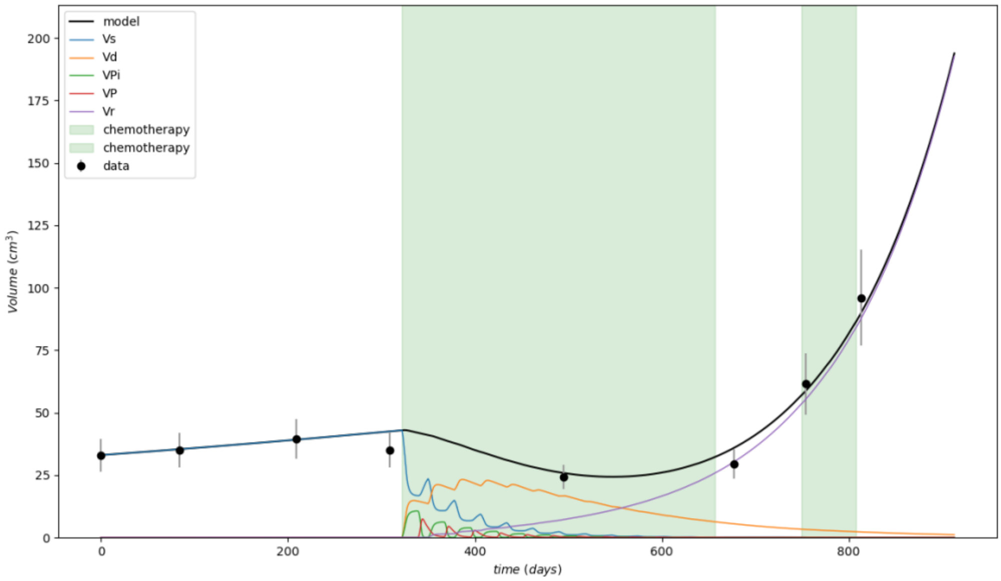

ρ_1	ρ_2	ρ_3	α_2	α_3	α_4	β	λ_1
0.00082	0.0079	0.007	0.04	0.0267	0.0476	0.128	1.5

Patient 5

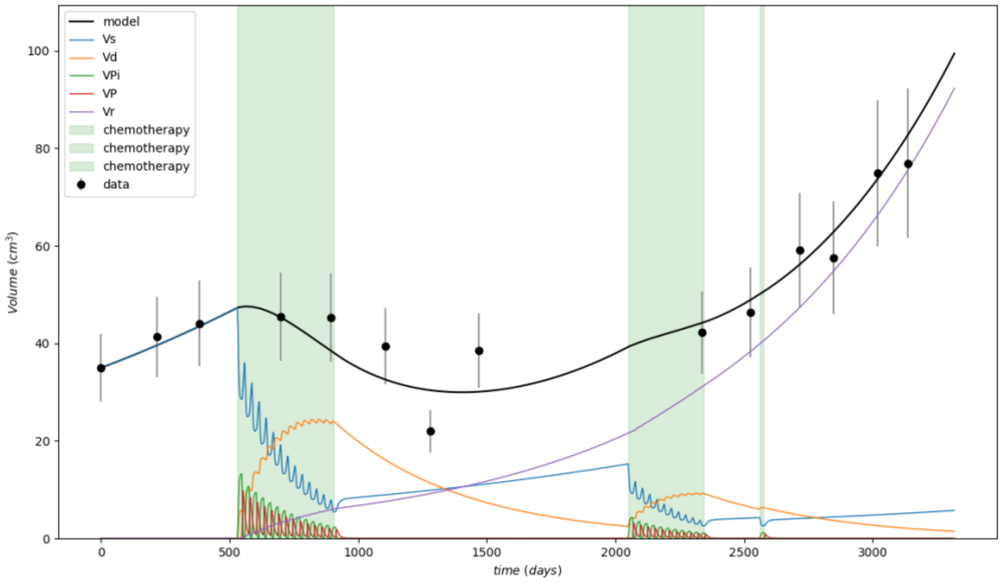

ρ_1	ρ_2	ρ_3	α_2	α_3	α_4	β	λ_1
0.00057	0.00111	0.0002	0.011	0.025	0.014	0.104	1.5

Patient 7

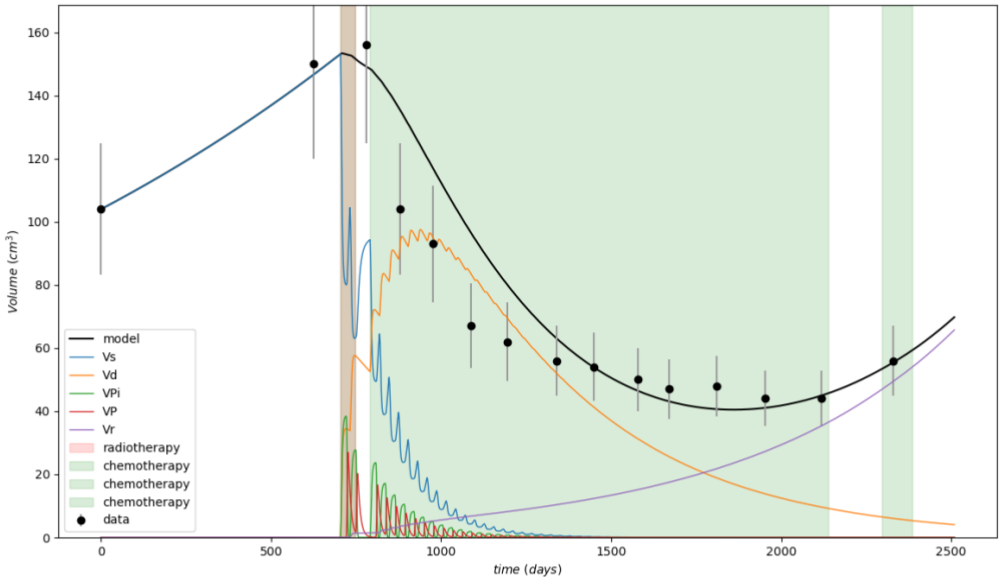

ρ_1	ρ_2	ρ_3	α_2	α_3	α_4	β	λ_1
0.00055	0.0016	0.00223	0.022	0.0239	0.0118	0.127	1.5

Patient 6

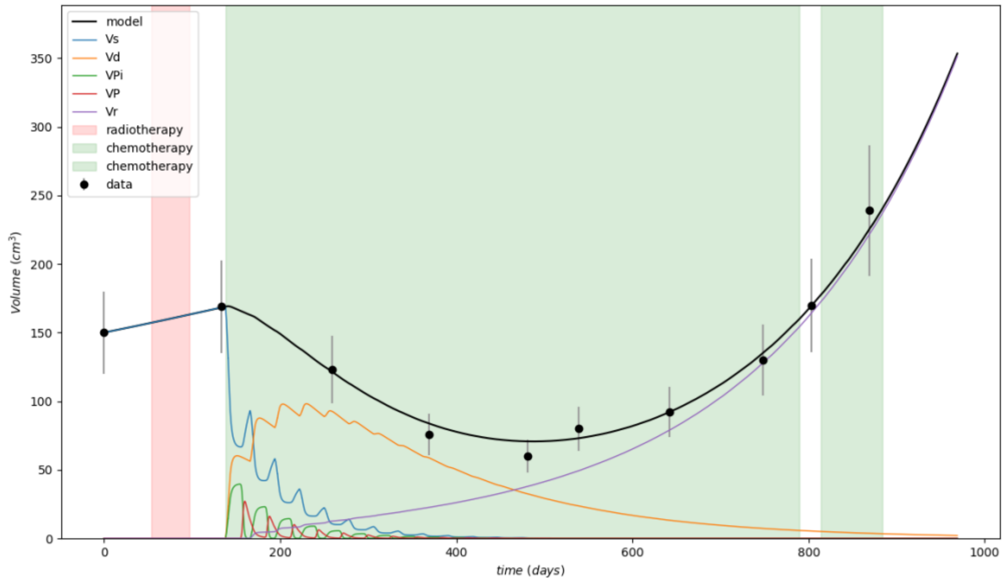

$\boldsymbol{\rho_1}$	$\boldsymbol{\rho_2}$	$\boldsymbol{\rho_3}$	$\boldsymbol{\alpha_2}$	$\boldsymbol{\alpha_3}$	$\boldsymbol{\alpha_4}$	$\boldsymbol{\beta}$	$\boldsymbol{\lambda_1}$
0.00086	0.004658	0.00575	0.04	0.0252	0.05	0.137	1.5
