# Supplementary material for: Overcoming chemotherapy resistance in low-grade gliomas: A computational approach
Source: PLoS Comput Biol. 2023 Nov 20;19(11):e1011208. doi: 10.1371/journal.pcbi.1011208 (PMC10695391; doi:10.1371/journal.pcbi.1011208)
Supplement: Attachment — Submitted filename: Review.pdf [file pcbi.1011208.s014.pdf]

Review of the article : Overcoming chemotherapy resistances in low-grade gliomas: a computational approach, by T. Delobel et al.

This article deals with a very important topic in oncology: the appearance of resistance to chemical treatment. In this article, the authors study the impact of the existence of a persister cells that can become resistant if another dose of chemotherapy is provided. They propose a mathematical model, fit clinical data from 7 patients and design virtual trials with different timing of the drug administration (more precisely different time spacing between drug doses) in order to test the one that optimizes survival and tumor reduction. The article is interesting and well-written. The study is original, and take into account significant biological insight. The methodology is rigorous. However, I have several questions that the authors should address.
The main criticism is about the fit of the 7 patients:

- Patient 2 and 4: there are no data point before the beginning of the treatment: how can you fix the value of the ρ_1 parameter?
- The author say that they fix the value of β to 0.1^{-1} day from a preliminary fit; please provide this preliminary fit.
- Are there enough data points to fix the values of the 8 parameters of the model without any ambiguity (or even 7 parameters if β is fixed)?
- What was the method used to ensure the robustness of the fit? Did you vary the initial value of the parameters used to find the best fit?
- Patient 7: it seems that the sensitive population is transformed into a damaged population very fast (see Fig3 G); the value of the parameter should be as large as for Patient 1 (see Fig3 A). How it comes that this parameter is not larger for this patient than for the other patients (except Patient 1), see Table 1?
- The authors say that they neglect the effect of radiotherapy for patients 6 and 7: how can they be sure that the observed decrease of the tumor's radius is not due to radiotherapy (Fig3)?
- For Patient 7, the authors say that they fitted only the eight last points: why 8 and not 7 or 9 (Fig3)? Why not all data points?

My other questions are bout the function f(E) used in the model.

- The authors explain that they use a function f(E) so that the recently created persister cells do not transform directly into resistant cells. However, it seems that the function f(E) is artificial: what biological argument could justify the fact that the persister cells become resistant when the second dose is low (and not high)? Why would the resistance happens at the second dose and not at the third, or even later?
- It seems that the main conclusion of the paper (the protocol with individual doses spaced 7 to 14 days apart would give the best treatment outcome, in terms of tumor reduction and survival of the patient) strongly depends on the value of β. How this conclusion changes with the value of β ? and how the same conclusion depends on the details of the function f(E) ? How do different values of the threshold (here set to E=0.05) in f(E) affect the results?

Other points:

- ref 57: please cite the article that has been published, instead of the preprint.
- Legend of Fig1: […] some sensitive cells are damaged […]
