# Supplementary material for: Overcoming chemotherapy resistance in low-grade gliomas: A computational approach
Source: PLoS Comput Biol. 2023 Nov 20;19(11):e1011208. doi: 10.1371/journal.pcbi.1011208 (PMC10695391; doi:10.1371/journal.pcbi.1011208)
Supplement: Attachment — Submitted filename: Reply_Letter_PCOMPBIOL-D-23-00817.pdf [file pcbi.1011208.s016.pdf]

Author Reply of Manuscript **PCOMPBIOL-D-23-00817** submitted to **PLOS Computational Biology**
Title: *Overcoming chemotherapy resistance in low-grade gliomas: A computational approach*
Authors: Thibault Delobel, Luis E. Ayala-Hernández, Jesús J. Bosque, Julián Pérez-Beteta, Salvador Chulián, Manuel García-Ferrer, Pilar Piñero, Philippe Schucht, Michael Murek, and Víctor M. Pérez-García.

Editor Comments

Dear Dr Bosque,

Thank you very much for submitting your manuscript "Overcoming chemotherapy resistances in low-grade gliomas: A computational approach" for consideration at PLOS Computational Biology.

As with all papers reviewed by the journal, your manuscript was reviewed by members of the editorial board and by several independent reviewers. In light of the reviews (below this email), we would like to invite the resubmission of a significantly-revised version that takes into account the reviewers' comments.

We cannot make any decision about publication until we have seen the revised manuscript and your response to the reviewers' comments. Your revised manuscript is also likely to be sent to reviewers for further evaluation.

When you are ready to resubmit, please upload the following:

[1] A letter containing a detailed list of your responses to the review comments and a description of the changes you have made in the manuscript. Please note while forming your response, if your article is accepted, you may have the opportunity to make the peer review history publicly available. The record will include editor decision letters (with reviews) and your responses to reviewer comments. If eligible, we will contact you to opt in or out.

[2] Two versions of the revised manuscript: one with either highlights or tracked changes denoting where the text has been changed; the other a clean version (uploaded as the manuscript file).

Important additional instructions are given below your reviewer comments.

Please prepare and submit your revised manuscript within 60 days. If you anticipate any delay, please let us know the expected resubmission date by replying to this email. Please note that revised manuscripts received after the 60-day due date may require evaluation and peer review similar to newly submitted manuscripts.

Thank you again for your submission. We hope that our editorial process has been constructive so far, and we welcome your feedback at any time. Please don't hesitate to contact us if you have any questions or comments.

Sincerely,

Jennifer A. Flegg Academic Editor PLOS Computational Biology

James O'Dwyer Section Editor PLOS Computational Biology

Dear Prof. Flegg and Prof. O'Dwyer,

We have read in detail the report on our manuscript. First of all, we would like to sincerely acknowledge the evaluators for their time, careful reading of our manuscript and for providing constructive criticism and valuable suggestions to improve the content of our work. We found them to be very helpful and below we detail our responses to the comments together with the changes incorporated into the manuscript that we now resubmit to *PLOS Computational Biology*.

Please, notice that, following the suggestion of one of the reviewers, we have decided to slightly change the title of our manuscript to "Overcoming chemotherapy resistance in low-grade gliomas: A computational approach".

Moreover, following the suggestion of the journal, we have also uploaded the original code used in our calculations to a public repository. This is now publicly available at `https://github.com/ThibDelo/LGG_resistance`. Finally, we have assigned a DOI to this code (`https://doi.org/10.5281/zenodo.8387519`).

The changes made in the revised manuscript have been highlighted in blue to facilitate their location. We now proceed to tackle all of the questions and suggestions raised by each of the reviewers.

Reviewer 1

Comment: *The present manuscript is a very detailed study of the mechanisms that lead low grade gliomas to resistance to the drug Temozolomide (TMZ), which, as far as I know, is the only drug able to pass the blood-brain barrier, and thus the drug on which one has to focus to understand pharmacoresistance in brain tumours. The study involves data recorded on patients, with their TMZ pharmacokinetic characteristics, and the construction of a population of virtual patients on these grounds. From the point of view of the study design, it is well documented and convincing.*
Reply: We thank the reviewer for his positive general assessment of our manuscript and for appreciating the useful insights it conveys. Below we address the specific comments and questions raised.

Comment: *However, I have real concerns about the ODE model, in particular as regards the subpopulation of resistant cells (V_R). It is not all clear to me, if this cell population is characterised by a strongly established resistance to TMZ, in a dead-end compartment, as it appears in the equations, whether it is due to an epigenetic (and thus in principle reversible, as in the case of EMT) change of phenotype ('from proneural to mesenchymal transition') or to a genetic mutation. If this resistant phenotype is fixed in such a way that no reversal is possible, by what mechanism is it fixed? If it is through an epimutation, then it should be reversible, be it only in the long term only. If it is through a genetic mutation, then equation (5) of the system makes sense, but such mutation should be searched for! By the way, the counter-intuitive (to me, if resistance is due to a costly non genetic mechanism, rather than to a thriftier established mechanism due to a forced - by long exposure to the drug - genetic mutation) mention of the fact that $\rho_2 > \rho_1$ would also make sense in the case of the second hypothesis.*
Reply: We appreciate the possibility that the reviewer offers us to better explain the underlying biological assumptions of our model. We next explain the details of the reasoning behind our work.

The development of TMZ resistance involves a complex interplay of numerous molecular mechanisms. These include epigenetic mechanisms, such as the methylation of the CpG island at the MGMT gene promoter, and also genetic mechanisms, such as the loss of function in mismatch repair (MMR) genes, the overexpression of ATP-binding cassette (ABC) transporters (Singh Tomar, 2021 *Biochim. Biophys. Acta - Rev. Cancer*), or the activation of ataxia telangiectasia mutated kinase (ATM) (Nogueira, 2011 *Oncotarget*; Singh, 2021 *Cancer Drug Resist.*). Other effects, like gene regulators of autophagy as ATG9B, also have an effect in acquired TMZ resistance (Yun, 2020 *Cell Death Dis.*), as does the vesicle-associated membrane protein 8 (VAMP8), which significantly increases cell proliferation and TMZ-resistance (Chen, 2015 *Neurooncol.*), in agreement with our results regarding $\rho_2 > \rho_1$. Moreover, MMR defects due to TMZ exposure have been found to lead to hypermutation in recurrent gliomas (Touat, 2020 *Nature*), which shows a transformed genetic landscape as a consequence of the treatment. Therefore, as a consequence of the multiple mechanisms involved in the acquisition of TMZ resistance, and the fixed nature that many of them share, that phenomenon must be irreversible. Taking together all these evidences, we chose to model the resistant compartment V_R as an irreversible state.

In order to clarify this point, we have now added this paragraph to the discussion section of our manuscript, strengthening the theoretical background that supports the formulation of our model and its results.

Comment: *The introduction of the intermediate compartments V_{PI} and V_P is natural and corresponds to the classical notions of drug-tolerant persisters and drug-tolerant expanded persisters, transposed from bacteriology to cancer biology in the princeps article of Sharma et al. in Cell 2010. The dead-end compartment V_R is something different from what is described in this princeps article. One could admit that strongly resistant cancer cells never die, being close to a stem cell state, but the unlimited exponential growth equation (5), even in the absence of stimulation by a drug, without any death nor fate reversal is intriguing, not to say that it is a big modelling problem.*

Reply: As the reviewer asserts, the evolution of the resistant population V_R is modeled as a proportional term that yields an exponential growth. Here, the proliferation rate ρ_2 is a net proliferation that accounts both for the intrinsic proliferation of the population as well as the death of resistant cells. However, as the balance between newborn and dying cells is positive, the net proliferation ρ_2 yields a positive growth term.

Notice that growth limitations could have been included in this equation, being the most traditional ways the addition of a logistic term $(1 - V/K)$ or a Gompertz term $(\log(K/V))$. In both cases, these terms set a limit to the overall size that the tumor can reach and have the additional cost of introducing a new parameter. Particularly, for the case of the logistic term, the results in the low volume range remain unchanged as the term vanishes for low V/K. This poses the problem of identifying the actual value of the carrying capacity K, as any arbitrarily big value of the parameter would yield results identical to those of a model that does not include this term; therefore, our model could be equivalently written in that way with unchanged results.

Moreover, the medical literature does not report cases of gliomas stopping their growth when approaching a hypothetical limiting size. In a study on 27 patients with *untreated* grade II gliomas, which excluded rapidly progressing patients that required earlier treatment, it was shown that the tumors grew continuously, with their diameter increasing linearly with time, that is, a cubic growth for their volume (Mandonnet, 2003 *Ann. Neurol.*). In a more recent study (Pérez-García, 2020, *Nat. Phys.*), a differential equation of the form $\dot{V} = aV^b$ was used to analyze the longitudinal growth of the volume V of low grade gliomas that received no treatment. The authors found that the value of the exponent b that best fitted the evolution was $b = 1.466 \pm 0.269$, what corresponds to an accelerated growth faster than an exponential.

Thus, we have opted here for a simple growth term which reduces the number of additional unidentifiable parameters and is compatible with the current knowledge regarding the continuous growth of gliomas. In order to clarify this, we have added a paragraph in our discussion.

Comment: *Mentioning this princeps and fundamental article by Sharma et al., I must say that I am surprised that the authors do not include it in their references, which may explain why they qualify as 'recently discovered' the existence of persister cells, while the Sharma paper is thirteen years old. Of course, the contexts of lung cancer cells exposed to high doses of drugs in a Petri dish and that of low grade glioma cells in the brains of patients are different. However, speaking of drug tolerant persister cells in cancer, given their reversal to sensitivity as described by Sharma et al., this article cannot be ignored.*

Reply: It was our intention to focus our discussion on the article that first reported the existence of persister cells during the acquisition of resistance to TMZ in gliomas, which serves as the basis for our research (Rabé, 2020 *Cell Death Dis.*).

However, we have no objection to citing previous references following the reviewer's suggestion. We have therefore added the reference to our manuscript and cited it in the

relevant places of the introduction and discussion.

Comment: *The fact that no reversal from the resistant cell population is prescribed in the model is to my meaning a bias of modelling which rules out the possibility of examining, with the help of these equations, adaptive therapy or so-called 'drug holiday' and 're-challenge' strategies that have been proposed by teams of clinicians, whereas the possibility of reversal of drug tolerant cell populations to a sensitive state, that might be included in the model, would make it possible.*

Reply: The reviewer is right to attach such a relevant value to the reversibility from drug-tolerant states to a sensitive state. In our work, the reversibility to a sensitive state of cells that are tolerant to drug plays a key role in our modeling strategy, equations and results. This is considered by means of an intermediate persistent state. However, according to the current corpus of knowledge, this state is only transient and is surpassed when exposure to the drug is maintained. This leads to a series of profound alterations that drift the cells from their normal behavior and render them invulnerable to the effect of TMZ. Citing Rabé, 2020 *Cell Death Dis.*, which serves as a biological inspiration for this rationale: "Cells gradually acquire an irreversible resistance to TMZ and reveal a transient state with a reduced proliferation rate, G2/M arrest, distinctive mitochondrial metabolism and reduced aggressivity in vivo." As a consequence, we have not considered any reversibility in the resistance state of LGG cells exposed to TMZ due to the lack of evidence regarding that transition.

Comment: *In summary, while I am favorably impressed by the sum of technical means presented to biologically and numerically investigate the question of resistance to TMZ in low grade gliomas, and to propose alternative therapeutical strategies, as compared to existing ones, I think that the ODE model is weak and highly questionable. To what extent it may fit or not to biological and clinical data cannot be an answer, not more than Ptolemy's armillary sphere to justify pre-Copernican Earth-centred visions of the cosmos in astronomical calculations, right though they might have seemed, before the Copernican revolution. And if the ODE model presented in the present manuscript is not simply false, perhaps due to particularities of low grade gliomas exposed to temolozomide, then it must be fully biologically justified (and at least somewhat modified, for instance by at least putting a logistic or Gompertz-like growth term in equation (5)).*

For these reasons, my evaluation is: to be resubmitted after major revision.

Reply: In the previous discussion we have addressed all the reviewer's objections. We hope that the deeper explanation of the biological background that we are providing in the revised version of the manuscript will reassure them of the validity of our modeling perspective or, at least, the reasoning supporting the modeling decisions.

This finishes our response to the comments of Reviewer 1.

Reviewer 2

Comment: *In this manuscript, the authors present an ordinary differential equations model of low-grade glioma (LGG) growth, treatment with temozolomide (TMZ), and relapse due to treatment resistance. They build on previous modeling work from their group by incorporating a recently documented intermediary to resistance, namely "persiste" cells, into the cancer growth model. The authors extracted longitudinal tumor volume data from 7 LGG patients treated with TMZ across 3 institutions in Europe and used these measurements to validate their model. They compared alternative treatment plans against the standard clinical protocol, assessing the impact on overall survival time, tumor reduction, and toxicity. The manuscript is well-organized, the figures are clear, and the work will likely be of interest to readers of PLOS Computational Biology. I raise several issues below, and include minor comments intended to improve the clarity and presentation of the work.*

Reply: We thank the reviewer for their time reviewing our manuscript and their favorable evaluation. Below we address the specific issues and comments raised.

Medium issues:

Comment: *1. The model assumes that drug efficacy is proportional to the drug concentration. Please add relevant citations like PMID: 30925722, but for LGG, to justify using a linear dose-response relationship for TMZ pharmacodynamics.*

Reply: We have added to our 'Materials and methods' section the reference suggested by the reviewer and an additional one reporting the same linear effect of TMZ concentration of glioblastoma cells. In vitro studies of glioma cell lines are much more often performed on glioblastoma cells than in low grade glioma cells and therefore this kind of pharmacodynamics studies are more likely to be carried out on the former. We assume here that the behavior is the same, as we are studying the same progenitor cell and the same drug.

Comment: *2. The assumptions underlying the authors' choice of activation function, $f(E)$, require biological justification or a more thorough discussion of the models limitations. Though it accomplishes the authors' desired effect with intermittent doses, having high concentrations of drug prevent transition to the persistent state while lower concentrations permit transition seems directly at odds with the authors' statement in line 23-24 of the introduction. If there was continuous uptake of the drug over a prolonged time period, presumably cells would still transition to the resistant state. Similarly, biological justification for why the transition time to the fully persister phenotype must be lower than one day is missing. Rather, it is the form of the activation function that forces the authors to make this assumption. Is it possible to achieve the desired delay/distribution of transition times from $V_P I$ to V_P by using a series of intermediate compartments, V_{P0}, V_{P1}, ..., V_{Pn}, V_P instead?*

Reply: The reviewer is right in highlighting the shortcomings of our explanation of the modeling perspective regarding the formation of persisters, and also the limitations that could arise when a different uptake of the drug is applied.

With regard to the apparent contradiction with line 23-24, notice that the transition to the resistant state is modeled here as a three steps process, with the formation of persisters accounting for the first two. In the first stage, the formation of intermediate persisters (V_{PI}) is motivated by—potentially high—concentrations of TMZ. Thus, it is the exposure to the drug that motivates the initiation of the process. Then, all these

intermediate persisters that have been formed must decay to the fully persister state (V_P), therefore, both persister compartments should be understood as persister cells that are created as a consequence of TMZ application. However, the formation of resistant cells is a process that takes time and, assuming the classical daily-dose application of TMZ, repeated doses. This is where the activation function comes into play, in the way that the cells that are ready to become fully resistant under exposure to TMZ are only formed when the previous application has finished. In order to resolve the apparent contradiction with line 23-24, we have chosen to rewrite the explanation of the 'Persister cells' compartment included in our 'Materials and methods' section.

The reviewer is right in pointing out that this modeling approach would fail if there was continuous uptake of the drug over a prolonged time period. In this sense, our model is restricted to the case of TMZ, which is administered in pills, with no less than a day between subsequent administration, and has a half-life of approximately 2 hours. We have now added this warning to the explanation of the activation function $f(E)$ in 'Materials and methods'. The same explanation applies for the justification of a transition time to the persister compartment lower than a day: this is the minimum expected time between doses, so at the time of application of a subsequent TMZ dose, the formed persisters should be already free to go back to sensitive or well transit to resistant as a function of the new applied dose concentration, that is, they should belong to the V_P compartment. We have also added this clarification to the S2 File.

Finally, the reviewer is right with regard to alternative and more general implementation of the same behavior. In this case, given the particularities of TMZ, we have chosen an approach that minimizes the number of additional populations and reduces the complexity of the model. We have also included the reviewer's proposal in the main text of our manuscript.

Comment: *3. The estimation of the number of individuals to enroll in a clinical trial or discussion of this result should be revised to account for the exclusion criteria used when selecting data for model parameter estimation. The authors considered data from a total of 91 patients initially, but only the 7 individuals who showed evidence of acquired resistance to TMZ treatment and did not receive other effective interventions were included in the study. This seems reasonable when modeling acquired resistance to TMZ, but could mean that a higher number of individuals would need to be enrolled in a clinical trial for the desired effect to be evident.*

Reply: In this work we use data from a retrospective study. These type of studies are characterized by a high rejection rate, as all the available data from the health institution is initially collected, but the information undergoes a subsequent filtering process in which only the patients with the desired characteristics are maintained.

In contrast, on a prospective study such as a clinical trial, the required variables are designed a priori, and every selected patient will respond to the same fixed inclusion criteria. In this work we suggest the minimum number of selected patients in the prospective study that would be needed for a the clinical trial to be conclusive, without regards to the patients that do not enter the trial for not fulfilling the requirements. We have added a sentence to that subsection remarking the reviewer's observation.

Minor issues:

1. *Title: Overcoming chemotherapy* **resistances** *in low-grade gliomas: A computational approach*

Reply: We thank the reviewer for spotting this mistake that has already been corrected in the revised version of our manuscript.

2. In general, the manuscript would benefit from careful reading for minor typos and precision of language. For example, using "what" instead of "which," "fitted" instead of "fit."
Reply: These typos have been now corrected.

3. Several paragraphs would benefit from reorganization to include a topic sentence that states the main point of the paragraph—for example, the paragraph starting on line 86 and the paragraph starting on line 509.
Reply: We have now added topic sentences to those paragraphs.

4. Line 155: "We considered that the one that best explains the LGG clinical response to TMZ observed in patients by Ricard et al. [28] is the mitotic catastrophe..." How was this determined? Is it important to the model results that the cell death is mitotic catastrophe? How would the model differ if fast-acting modes of cell death were included?
Reply: The main consequence of cell death by mitotic catastrophe is a time delay existing between the application of the therapy and the actual death. This way, cells get an irreparable damage but it is not until the cell attempts division that this damage leads to the death of the cell. Therefore, the consideration of this way of death is included in the model by the inclusion of a *damaged* compartment that contributes to the volume until the cells later die. Alternatively—or additionally—a direct cell death term could be considered to model fast-acting modes of cell death. Should this kind of term replace the current death term, the sustained dynamical effects of the drug would disappear and all the effect of the therapy would happen in a short period of time, what would reduce the adequacy of the model (see, for instance, Pérez-García, 2014 *Math. Med. Biol.*). We have now added a short sentence explaining this within the presentation of the damaged cells compartment.

5. Line 266-267: "The number of TMZ cycles administered to the virtual patients was drawn from a discrete normal distribution with a mean of 19 cycles and a variance of 7, excluding values lower than 6 and higher than 34 to avoid unrealistic values (S1 FigB)." Please clarify, why is the number of TMZ cycles drawn from a distribution? Is this not set by the trial protocol?
Reply: The reviewer is right. In our analysis we intended to get as close as possible to the real situation represented by our patient's dataset, where every patient happened to receive a different number of cycles. For that, we generated virtual cases with different number of cycles in agreement with the distribution shown by the real data.

However, making a proposal for a new protocol and testing it in a clinical trial would imply recruiting a number of patients for each arm (100 for the virtual trials performed here) and delivering a pre-fixed number of cycles distributed according to standard protocol in one case, and the proposed one in the other. Therefore, in addition to the previous analysis, we have now included new clinical trials setting the number of cycles in the protocol and testing the difference in overall survival of the ID protocols against the classical one.

This new analysis has given rise to a new subsection in 'Results' and a new supplementary figure (new S7 Fig), as well as modifications in 'Materials and methods'.

6. Table 1: The authors could improve readability by using scientific notation for parameter values and including a row annotating what each parameter means so that reader does not have to scroll back and forth.

Reply: We have now adapted that table for better readability.

7. Figure 3: For Patient A, the final data point is not in agreement with the model prediction. Could this have to do with the radiotherapy, which is not being modeled? The authors assert that for these patients, data did not show radiotherapy to be effective, but perhaps instead this should be discussed as a limitation of the work/potential motivation for modeling combination therapy.

Reply: This disagreement between the data and the prediction is most probably due to the effect of radiation therapy. It was not our intention to assert that radiotherapy was not effective, therefore we have included brief commentaries highlighting the potentially important effect of that unmodeled treatment and removed an ambiguous sentence from the discussion that could mislead the reader.

We have also added a sentence to the discussion indicating the relevance of a combined study of radiotherapy and chemotherapy. As a matter of fact, this is the objective of the project TED2021-132318B-I00 funded by the Spanish Government which is right now being carried out.

8. Figure 3: Missing error bars on patient 7?

Reply: We only included error bars for patients that have been measured by means of the ellipsoid approximation. Patient 7 was measured by a precise segmentation carried out slice by slice and therefore considered much more precise, so error bars were omitted. We have added a sentence to the caption of Fig 3 explaining this fact.

9. Lines 347-349: "Similar dynamics were observed for other patients..." I suggest listing some summary statistics for this result. What was the average threshold and standard deviation? Is 42 days an outlier?

Reply: We have now added the mean number of days between doses that causes loss of control (55 days) and the standard deviation (17.7 days).

10. Line 354: "Our results thus showed that spacing out each single dose between 7 and 42 days increases both OS and tumor shrinkage for all selected patients of our dataset." The increased tumor shrinkage beyond that attained by the standard protocol is not clear for the larger dose intervals. Perhaps indicating the lowest tumor size with a horizontal line on figure 4 or using a discrete color key instead of the continuous color bar would be sufficient. An alternative would be plotting maximum tumor volume lost vs. overall survival for the different dosage plans.

Reply: As the reviewer suggested, we now have used a discrete color key for Fig 4, and plotted a horizontal line indicating the lowest tumor size. We have added a short sentence to the caption explaining the new line, but have opted for skipping further details in order to keep the focus on the key points of the paper.

11. Line 380-381: "To ensure control of tumor growth on every patient, ID protocols in which at least one patient experienced loss of control were neglected." This requires further clarification. Did the 42 day maximum rest interval hold for the virtual cohorts?

Reply: This was an omission in the original manuscript. For the 20 clinical trials performed for ID35 protocol and also for ID42 protocol, there was at least one patient in which there was a loss of control in the total volume. Therefore, these protocols were neglected.

We have reorganized the information on that subsection and made it clear that we are disregarding those two protocols in favor of preserving the safety of all the enrolled patients.

12. Line 393-394: "...spacings like ID14 and ID21 provide a better compromise between OS gain and reduction of tumor volume." The authors should add justification for why this is the right balance for this trade-off. Perhaps cite literature around tolerable tumor burden to justify this.
Reply: This question builds on the previous one. We chose these two protocols because they show safety in controlling tumor volume while at the same time providing significant improvements in terms of overall survival. Additionally, they have the advantage of their easy clinical implementation, since they propose giving a dose regularly every two weeks, or every three weeks, respectively. We have now added this explanation to that subsection.

13. Line 402: 20 independent clinical trials? If not, what does initial mean in this context?
Reply: We thank the reviewer for spotting this mistake that has been already corrected in the revised version.

14. Line 415: "Adaptive administration of TMZ may limit toxicity but would not improve survival" I suggest revising this section title to make it clear that the adaptive plan tested here DOES improve survival over the standard cyclic protocol, just not beyond the gains from individual dosing on a fixed schedule.
Reply: We have changed the title to "Adaptive variations of ID protocols can be used to limit toxicity".

15. A key idea behind adaptive therapy is that there is a fitness cost to resistance, so that the therapy-sensitive population can outcompete the therapy-resistant population in the absence of treatment. Without this relationship, removing therapy does not control the resistant population. Because of the model assumption that TMZ resistant cells gain fitness, it seems out of place to consider adaptive therapy for long-term control. It would be good for the authors to explain this context a little more fully at the start of the section on adaptive therapy or in the introduction. The authors have shown that using fewer doses than the ID21 and ID 14 plans prescribe can be equally effective, even when including a long break in therapy. I would encourage framing these results as allowing a patient a drug holiday to reduce side effects.
Reply: With regard to the first part of this comment, we have included the relevant comment of the reviewer in our discussion regarding the adaptive variation of the ID protocols shown here and have elaborated on the possibilities of such a case.

Regarding the second part, we have also reframed our results and discussion to highlight the benefits that merging adaptive therapy with ID protocols may have for the patients.

16. If sticking with the adaptive therapy framing, I was also curious as a reader, what happens as the screening interval changes?
Reply: We have performed additional simulations with lower screening times (1 month) and higher screening times (6 months), whose results are now shown on the new S8 Fig. Even though there is a slightly lower survival for the case of a longer time between screenings, the difference is not very high. This happens because the sampling time in

all cases is small with respect to the periods in which the therapy application is active or inactive. We have now added this brief explanation to the main text.

17. In line 565, "Our results demand to be validated in a real world implementation," reads as rather aggressive. I suggest toning this language down.

Reply: We have changed that sentence to "These in silico results pave the way for potential future trials that may result in improvements in the survival and quality of life of LGG patients. Our study suggests specific variations with an easy clinical implementation that could raise the efficacy of an approved drug like TMZ."

Suggestions:

1. The work would be strengthened by placing the model directly in conversation with previous modeling results. Quantifying the extent to which including the persistent cell population improves model fits and noting whether including the persisters changes the optimal treatment plan would enhance the novelty and/or robustness of the results. What happens if you test a combination scheme, as suggested in previous work from the group (`https://doi.org/10.1371/journal.pcbi.1006778`), with this model framework?

2. If the authors are able to quantify how many fewer doses/more rest days patients would get with the proposed protocol compared with the 5 days on, 23 days off standard protocol, this would provide further motivation to test these treatment plans in practice. Especially if the duration of rest can be linked to a reduction in side effects.

3. Figure 1: Panel A would be more informative if the authors indicate how the tumor volume is changing at these different stages as well.

4. Figure 2: The authors could emphasize the differences between these plans by indicating the magnitude and duration of side effects, perhaps with shading.

5. Figure 4: It could be informative to add a second panel showing the resistant population under each treatment plan.

6. Figure 5: This is an important figure to include in the work, but perhaps could be moved to the supplement.

7. Figure 6: Nice visualization of key result. This could be condensed into one panel instead of 4.

8. Using the phrase "spaces" to refer to the rest period between TMZ doses reads as too informal to me. Unless this is a technical term, perhaps use the phrase dosing interval or rest period instead of "spaces."

Reply: We thank the reviewer for their suggestions. We have extended our discussion regarding previous modeling results and, in particular, the previous work from the group (Pérez-García, 2019 *PLOS Comp Bio*) in the first and ninth paragraphs of the discussion. We have also highlighted the fact that we are comparing here a different way of distributing the doses, but have not reduced the number of overall doses. For that, in our in silico trials we initially used a random number of doses that was in correspondence with the doses that the real patients have received. Now, following the reviewer's suggestion we have also included clinical trials for which a pre-defined protocol is used and every patient is assigned the same number of drug cycles. Even in the case of adaptive therapy, the saved doses are thought to use later on when the tumor volume is big enough to restart the treatment. In our Fig 1, we have now modified the first panel to give a sense of how

the tumor volume changes at the different stages of the disease. Finally, we have moved Fig 5 to Supporting Information (now new S4 Fig) and have changed the phrase "spaces" to the more formal phrases "dosing interval" and "rest periods".

This finishes our response to the comments of Reviewer 2.

Reviewer 3

Comment: *This article deals with a very important topic in oncology: the appearance of resistance to chemical treatment. In this article, the authors study the impact of the existence of persister cells that can become resistant if another dose of chemotherapy is provided. They propose a mathematical model, fit clinical data from 7 patients and design virtual trials with different timing of the drug administration (more precisely different time spacing between drug doses) in order to test the one that optimizes survival and tumor reduction. The article is interesting and well-written. The study is original, and take into account significant biological insight. The methodology is rigorous. However, I have several questions that the authors should address.*

The main criticism is about the fit of the 7 patients:

Reply: We thank the reviewer for their good overall assessment of our work and above we tackle the specific issues commented by them.

Comment: *Patient 2 and 4: there are no data point before the beginning of the treatment: how can you fix the value of the* ρ_1 *parameter?*

Reply: Even though there is no data to fit the pre-treatment behavior in terms of tumor growth, these parameters are implicit in the *overall growth+response to the treatment* seen in the time evolution. For instance, there is a correlation between response to treatment and the growth rate of the sensitive population. The method that we used for all the patients was fitting the overall time evolution arising from the simulations (given that the treatment is known, and so it is its implementation in the model) to the specific data points that we had. Therefore, we did not distinguish different regimes for specific parameters' fits, but used a single and general time range, taking advantage of the appropriate inclusion of the treatment that is taken care of by the model.

We have now added a short sentence explaining this to the "Estimation of patient-specific parameters" subsection.

Comment: *The author say that they fix the value of* β *to 0.1 day*$^{-1}$ *from a preliminary fit; please provide this preliminary fit.*

Reply: These preliminary results have now been added to the Supporting Information as S3 File and referenced in the main text.

Comment: *Are there enough data points to fix the values of the 8 parameters of the model without any ambiguity (or even 7 parameters if* β *is fixed)?*

Reply: Our model has a total of 6 free parameters (or 7 if β is included). Ideally, it would be good to build a model of LGG growth and response to TMZ that had even less parameters, however, that would limit our ability to represent the underlying biology. Moreover, notice that 8 is the minimum number of data points in the patients' data and all the other patients have a higher number of time follow-ups up to a maximum of 15. For all seven patients, our model achieves a reasonable fit, representing well not only the time evolution of the disease but also the treatment response.

We have now added a sentence highlighting the shortcoming pointed out by the reviewer of having 6 parameters to the paragraph of our discussion that deals with the parameters and fitting.

Comment: *What was the method used to ensure the robustness of the fit? Did you vary the initial value of the parameters used to find the best fit?*

Reply: The fits were implemented as a numerical optimization process by means of the Python function `minimize` from the SciPy package. To ensure the robustness of the fit, several initial random seeds within the prescribed bounds were used. This raises the probabilities that the result of the optimization process is a global minimum (best fit), rather than a local minimum. From those different fits corresponding to different seeds we selected the fit showing a smaller mean squared error.

We have now added this further explanation to the “Estimation of patient-specific parameters” subsection of our manuscript.

Comment: *Patient 7: it seems that the sensitive population is transformed into a damaged population very fast (see Fig3 G); the value of the parameter should be as large as for Patient 1 (see Fig3 A). How it comes that this parameter is not larger for this patient than for the other patients (except Patient 1), see Table 1?*
Reply: In terms of slope, the transformation of sensitive cells in damaged cells is much steeper in patient 1 than in patient 7. That is the reason why the parameter ψ ruling that transition is higher for the former. A graphical way to see this is noticing the ripples from the evolution of damaged cells, which indicate given doses of the treatment: For patient 1 only three doses are enough to generate the proportional damage of 6 doses in patient 7. Also, the time scale is much shorter for patient 1.

Comment: *The authors say that they neglect the effect of radiotherapy for patients 6 and 7: how can they be sure that the observed decrease of the tumor's radius is not due to radiotherapy (Fig3)?*
Reply: The reviewer is right. With the current information, we can only hypothesize what the effect of this treatment is. For both patients, there is a growth in tumor volume without regard to the application of radiotherapy. These encouraged us to keep the data and focus on the chemotherapy application, even though, as the reviewer suggests, the effect of radiation cannot be disregarded. It was not our intention to assert that radiotherapy was not effective, therefore we have included brief commentaries highlighting the potentially important effect of that unmodeled treatment, removed an ambiguous sentence from the discussion that could mislead the reader, and removed the word “neglect” from the presentation of our results.

Actually, it would be very interesting to also consider radiation therapy in our approach and study the relevance of a combined study of radiotherapy and chemotherapy. We have added a short sentence to the discussion highlighting the importance of that work. As a matter of fact, we have a current project funded by the Spanish Government (TED2021-132318B-I00) that tackles that issue an is right now being carried out by our team.

Comment: *For Patient 7, the authors say that they fitted only the eight last points: why 8 and not 7 or 9 (Fig3)? Why not all data points?*
Reply: We clearly used a misleading sentence here. In order to reinforce justification of the inclusion of the data from patient 7, without regard to the radiation therapy that the patient had undergone, we asserted that the obtained fit is good once the possible effect of that treatment is more likely to have vanished. That is why we referred to the—good—fit of the last eight points.

Comment: *My other questions are bout the function f(E) used in the model.*

The authors explain that they use a function $f(E)$ so that the recently created persister cells do not transform directly into resistant cells. However, it seems that the function

$f(E)$ is artificial: what biological argument could justify the fact that the persister cells become resistant when the second dose is low (and not high)? Why would the resistance happens at the second dose and not at the third, or even later?
Reply: Indeed, the function $f(E)$ is a mathematical implementation that tries to simplify the complex underlying biology. With regard to why persisters are allowed to pass to the V_P compartment at low doses, notice that the transition to the resistant state is modeled here as a three steps process, with the formation of persisters accounting for the first two. In the first stage, the formation of intermediate persisters (V_{PI}) is motivated by—potentially high—concentrations of TMZ. Thus, it is the exposure to the drug that motivates the initiation of the process. Then, all these intermediate persisters that have been formed must decay to the fully persister state (V_P), therefore, both persister compartments should be understood as persister cells that are created as a consequence of TMZ application. However, the formation of resistant cells is a process that takes time and, assuming the classical daily-dose application of TMZ, repeated doses. This is where the activation function comes into play, in the way that the cells that are ready to become fully resistant under exposure to TMZ are only formed when the previous application has finished. In order to clarify this modeling choice, we have rewritten the explanation of the 'Persister cells' compartment included in our 'Materials and methods' section.

With regard to the second question, we are not claiming that resistance happens at the second dose. With the use of the switch function $f(E)$ we are rather setting a lower threshold for this transition to happen. There are alternative approaches that could be used to generate a similar behavior, but they would imply using more populations and additional parameters. We have now added this information to our exposition in 'Materials and methods'.

Comment: *It seems that the main conclusion of the paper (the protocol with individual doses spaced 7 to 14 days apart would give the best treatment outcome, in terms of tumor reduction and survival of the patient) strongly depends on the value of β. How this conclusion changes with the value of β? and how the same conclusion depends on the details of the function $f(E)$? How do different values of the threshold (here set to $E = 0.05$) in $f(E)$ affect the results?*
Reply: In order to ensure the robustness of our results to changes in the parameters, we performed new virtual clinical trials comparing the protocol ID21 to the classical C28. We maintained all the ranges of the parameters and overall procedure but changed the fixed value of β for each of the different trials. We carried out a total of 4 new trials to study the values $\beta = 0.02$ day^{-1}, $\beta = 0.05$ day^{-1}, $\beta = 0.5$ day^{-1} and $\beta = 1$ day^{-1}. These are wide variations around the original value $\beta = 0.1$ day^{-1}. The Kaplan-Meier curves showing the results of these trials are now the new S5 Fig of the Supporting Information. The results show that our conclusion holds for other values of the parameter β. Notice that higher values of the parameter β seem to be associated with higher survival benefit of the protocols that spaces out the doses, due to the higher pace at which the cells come back from an induced persister state to a sensitive state. As a consequence, even though we do not expect a high inter-patient heterogeneity for this parameter, if that was not the case, then the parameter β, i.e. the ability of the cells to go back to a sensitive state after exposure to the drug, could be used as an anatomical pathology biomarker that would discriminate which patients would benefit more from an ID protocol. We have now added the results to the Supporting Information and have added a brief discussion on the implications to the main text of the manuscript.

With regard to the details of the function $f(E)$, we have followed the same procedure

by running two new clinical trials in which only the value of the threshold parameter changed from the previous simulations. Thus, we have now used $f(E) = 7.5(1-\tanh((E-0.05)/0.05))$ for the first new trial and $f(E) = 7.5(1-\tanh((E-0.1)/0.1))$ for the second. In this case, we do not try lower values of the parameters ruling the switching function, as the initial one was already very low. The Kaplan-Meier curves showing the difference between a C28 protocol and an ID21 with these new parameters in the switching function are shown in the new S6 Fig. It is readily seen that this parameter does not change our conclusions in any way. We have now added these results to Supporting Information of our manuscript and commented on them briefly within the text.

Comment: *Other points:*

Ref 57: please cite the article that has been published, instead of the preprint.
Reply: We thank the reviewer for this update.

Comment: *Legend of Fig1: [...] some sensitve cells are damaged [...]*
Reply: We thank the reviewer for spotting this typo that has been now corrected.

This finishes our response to the comments of Reviewer 3.

This concludes our reply to Manuscript **PCOMPBIOL-D-23-00817**.

Yours sincerely,

The Authors

October 2, 2023
