# Supplementary material for: Overcoming chemotherapy resistance in low-grade gliomas: A computational approach
Source: PLoS Comput Biol. 2023 Nov 20;19(11):e1011208. doi: 10.1371/journal.pcbi.1011208 (PMC10695391; doi:10.1371/journal.pcbi.1011208)
Supplement: Attachment — Submitted filename: PCOMPBIOL_Review.docx [file pcbi.1011208.s015.docx]

PCOMPBIOL-D-23-00817

Overcoming chemotherapy resistances in low-grade gliomas: A computational approach

In this manuscript, the authors present an ordinary differential equations model of low-grade glioma (LGG) growth, treatment with temozolomide (TMZ), and relapse due to treatment resistance. They build on previous modeling work from their group by incorporating a recently documented intermediary to resistance, namely “persister” cells, into the cancer growth model. The authors extracted longitudinal tumor volume data from 7 LGG patients treated with TMZ across 3 institutions in Europe and used these measurements to validate their model. They compared alternative treatment plans against the standard clinical protocol, assessing the impact on overall survival time, tumor reduction, and toxicity. The manuscript is well-organized, the figures are clear, and the work will likely be of interest to readers of PLOS Computational Biology. I raise several issues below, and include minor comments intended to improve the clarity and presentation of the work.

**Medium Issues:**

1. The model assumes that drug efficacy is proportional to the drug concentration. Please add relevant citations like PMID: 30925722, but for LGG, to justify using a linear dose-response relationship for TMZ pharmacodynamics.
2. The assumptions underlying the authors’ choice of activation function, f(E), require biological justification or a more thorough discussion of the models limitations. Though it accomplishes the authors’ desired effect with intermittent doses, having high concentrations of drug prevent transition to the persistent state while lower concentrations permit transition seems directly at odds with the authors’ statement in line 23-24 of the introduction. If there was continuous uptake of the drug over a prolonged time period, presumably cells would still transition to the resistant state. Similarly, biological justification for why the transition time to the fully persister phenotype must be lower than one day is missing. Rather, it is the form of the activation function that forces the authors to make this assumption. Is it possible to achieve the desired delay/distribution of transition times from VPI to VP by using a series of intermediate compartments, V_P0, V_P1,…, V_Pn, VP instead?
3. The estimation of the number of individuals to enroll in a clinical trial or discussion of this result should be revised to account for the exclusion criteria used when selecting data for model parameter estimation. The authors considered data from a total of 91 patients initially, but only the 7 individuals who showed evidence of acquired resistance to TMZ treatment and did not receive other effective interventions were included in the study. This seems reasonable when modeling acquired resistance to TMZ, but could mean that a higher number of individuals would need to be enrolled in a clinical trial for the desired effect to be evident.

**Minor Issues:**

1. Title: Overcoming chemotherapy **resistance~~s~~** in low-grade gliomas: A computational approach
2. In general, the manuscript would benefit from careful reading for minor typos and precision of language. For example, using “what” instead of “which,” “fitted” instead of “fit.”
3. Several paragraphs would benefit from reorganization to include a topic sentence that states the main point of the paragraph—for example, the paragraph starting on line 86 and the paragraph starting on line 509.
4. Line 155: “We considered that the one that best explains the LGG clinical response to TMZ observed in patients by Ricard et al. [28] is the mitotic catastrophe…” How was this determined? Is it important to the model results that the cell death is mitotic catastrophe? How would the model differ if fast-acting modes of cell death were included?
5. Line 266-267: “The number of TMZ cycles administered to the virtual patients was drawn from a 266 discrete normal distribution with a mean of 19 cycles and a variance of 7, excluding 267 values lower than 6 and higher than 34 to avoid unrealistic values (S1 FigB).” Please clarify, why is the number of TMZ cycles drawn from a distribution? Is this not set by the trial protocol?
6. Table 1: The authors could improve readability by using scientific notation for parameter values and including a row annotating what each parameter means so that reader does not have to scroll back and forth.
7. Figure 3: For Patient A, the final data point is not in agreement with the model prediction. Could this have to do with the radiotherapy, which is not being modeled? The authors assert that for these patients, data did not show radiotherapy to be effective, but perhaps instead this should be discussed as a limitation of the work/potential motivation for modeling combination therapy.
8. Figure 3: Missing error bars on patient 7?
9. Lines 347-349: “Similar dynamics were observed for other patients …” I suggest listing some summary statistics for this result. What was the average threshold and standard deviation? Is 42 days an outlier?
10. Line 354: “Our results thus showed that spacing out each single dose between 7 and 42 days increases both OS and tumor shrinkage for all selected patients of our dataset.” The increased tumor shrinkage beyond that attained by the standard protocol is not clear for the larger dose intervals. Perhaps indicating the lowest tumor size with a horizontal line on figure 4 or using a discrete color key instead of the continuous color bar would be sufficient. An alternative would be plotting maximum tumor volume lost vs. overall survival for the different dosage plans.
11. Line 380-381: “To ensure control of tumor growth on every patient, ID protocols in which at least one patient experienced loss of control were neglected.” This requires further clarification. Did the 42 day maximum rest interval hold for the virtual cohorts?
12. Line 393-394: “… spacings like ID14 and ID21 provide a better compromise between OS gain and reduction of tumor volume.” The authors should add justification for why this is the right balance for this trade-off. Perhaps cite literature around tolerable tumor burden to justify this.
13. Line 402: 20 independent clinical trials? If not, what does initial mean in this context?
14. Line 415: “Adaptive administration of TMZ may limit toxicity but would not improve survival” I suggest revising this section title to make it clear that the adaptive plan tested here DOES improve survival over the standard cyclic protocol, just not beyond the gains from individual dosing on a fixed schedule.
15. A key idea behind adaptive therapy is that there is a fitness cost to resistance, so that the therapy-sensitive population can outcompete the therapy-resistant population in the absence of treatment. Without this relationship, removing therapy does not control the resistant population. Because of the model assumption that TMZ resistant cells gain fitness, it seems out of place to consider adaptive therapy for long-term control. It would be good for the authors to explain this context a little more fully at the start of the section on adaptive therapy or in the introduction. The authors have shown that using fewer doses than the ID21 and ID 14 plans prescribe can be equally effective, even when including a long break in therapy. I would encourage framing these results as allowing a patient a drug holiday to reduce side effects.
16. If sticking with the adaptive therapy framing, I was also curious as a reader, what happens as the screening interval changes?
17. In line 565, “Our results demand to be validated in a real world implementation,” reads as rather aggressive. I suggest toning this language down.

Suggestions:

1. The work would be strengthened by placing the model directly in conversation with previous modeling results. Quantifying the extent to which including the persistent cell population improves model fits and noting whether including the persisters changes the optimal treatment plan would enhance the novelty and/or robustness of the results. What happens if you test a combination scheme, as suggested in previous work from the group (<https://doi.org/10.1371/journal.pcbi.1006778>), with this model framework?
2. If the authors are able to quantify how many fewer doses/more rest days patients would get with the proposed protocol compared with the 5 days on, 23 days off standard protocol, this would provide further motivation to test these treatment plans in practice. Especially if the duration of rest can be linked to a reduction in side effects.
3. Figure 1: Panel A would be more informative if the authors indicate how the tumor volume is changing at these different stages as well.
4. Figure 2: The authors could emphasize the differences between these plans by indicating the magnitude and duration of side effects, perhaps with shading.
5. Figure 4: It could be informative to add a second panel showing the resistant population under each treatment plan.
6. Figure 5: This is an important figure to include in the work, but perhaps could be moved to the supplement.
7. Figure 6: Nice visualization of key result. This could be condensed into one panel instead of 4.
8. Using the phrase “spaces” to refer to the rest period between TMZ doses reads as too informal to me. Unless this is a technical term, perhaps use the phrase dosing interval or rest period instead of “spaces.”
